# The effect of hypoxia on the proteomic signature of pig adipose-derived stromal/stem cells (pASCs)

**DOI:** 10.1038/s41598-020-76796-7

**Published:** 2020-11-18

**Authors:** Joanna Bukowska, Mariola Słowińska, Patrycja Cierniak, Marta Kopcewicz, Katarzyna Walendzik, Trivia Frazier, Barbara Gawrońska-Kozak

**Affiliations:** 1 Institute of Animal Reproduction and Food Research, Polish Academy of Sciences, Tuwima 10, 10-748 Olsztyn, Poland; 2 LaCell LLC, New Orleans, LA USA; 3 Obatala Sciences Inc., New Orleans, LA USA

**Keywords:** Bioinformatics, Data mining, Data processing, Microarrays, Software, Quality control, RNA sequencing

## Abstract

Human adipose-derived stem cells (ASCs) have potential to improve wound healing; however, their equivalents from domestic animals have received less attention as an alternative cell-based therapy for animals or even humans. Hypoxia is essential for maintaining stem cell functionality in tissue-specific niches. However, a cellular response to low oxygen levels has not been demonstrated in pig ASCs. Hence, the goal of our study was to characterize ASCs isolated from the subcutaneous fat of domestic pigs (pASCs) and examine the effect of hypoxia on their proteome and functional characteristics that might reproduce pASCs wound healing ability. Analysis of immunophenotypic and functional markers demonstrated that pASCs exhibited characteristics of mesenchymal stem cells. Proteomic analysis revealed 70 differentially abundant proteins between pASCs cultured under hypoxia (1% O_2_) or normoxia (21% O_2_). Among them, 42 proteins were enriched in the cells exposed to low oxygen, whereas 28 proteins showed decrease expression following hypoxia. Differentially expressed proteins were predominantly involved in cell metabolism, regulation of focal and intracellular communication, and attributed to wound healing. Functional examination of hypoxic pASCs demonstrated acquisition of contractile abilities in vitro. Overall, our results demonstrate that hypoxia pre-conditioning impacts the pASC proteome signature and contractile function in vitro and hence, they might be considered for further cell-based therapy study on wound healing.

## Introduction

Adipose-derived stem cells (ASCs) are increasingly recognized as a vital cell source for cell-based therapy in regenerative medicine^1–3^. Having shown multiple advantages including easy collection, availability, weak immunogenicity, and self-renewal and multilineage differentiation capacities, ASCs are a potential therapeutic modality in a variety of diseases^4,5^. ASCs contribute to cutaneous wound healing in a variety of rodent wound models. Indeed, ASCs administration reduces inflammation, enhances re-epithelialization, and improves healing by promoting neovascularization in excisional wounds in normal and diabetic rats^6,7^. Furthermore, delivery of human ASCs to a murine pressure ulcer model accelerated wound healing by reducing inflammation and tissue hypertrophy, enhancing collagen deposition, and activating the expression of reparative genes (*Tgfβ, Pdgfβ, Vegf, Hgf, Mmp-9*, and *Mmp-13*)^8^. The mechanisms by which ASCs exert their effects on the wound microenvironment fall into two paradigms: differentiation of ASCs into specific cell types that replace defective cells^9^ and secretion of soluble factors that act in a paracrine manner to direct the healing process^10^. Of these, paracrine signaling has emerged as a critical pathway underlying ASCs therapeutic/pro-healing properties^11,12^.

Low oxygen (hypoxia) plays a critical role in the maintenance of stem cell characteristics as it is a fundamental component of stem cell niche in vivo^13^. The oxygen concentration in human adipose tissue oxygen concentration is < 3–4% indicating that ASCs naturally reside in relatively oxygen-deficient locations^14^. However, standard culture conditions of ASCs and various stem cells collected from mammalian tissues are under atmospheric oxygen concentration (20–21%), which does not represent their natural physiological condition. Therefore, exposing ASCs to low oxygen provides a more accurate environment. Growing evidence shows that hypoxia improves ASCs functions including survival, proliferation, migration, and differentiation capacities in vitro and in vivo^15–17^*.* Importantly, hypoxia increases ASCs paracrine activity, particularly with respect to promotion of angiogenesis via up-regulation of vascular endothelial growth factor (VEGF)^18^. Frazier *et al*.^19^ showed that pre-conditioning of human ASCs with low oxygen reduced secretion of extracellular matrix (ECM) proteins and type 2 cytokines (interleukin [IL]-6, IL-13, monocyte chemoattractant protein [MCP]-1, CD40 ligand) involved in the initiation of fibrosis. These results suggest that enhancing these ASCs characteristics with hypoxia may increase their regenerative potential and thus augment healing following cell delivery to the injured tissues.

The hypothesis that hypoxic pre-conditioning of ASCs provides functional enhancement^15,18,20^ and makes the cells more attractive for clinical translational science has encouraged an interest in characterizing the hypoxia-responsive characteristics of ASCs on the proteomic level^21^. This -omics technique might provide a tool for predicting ASCs safety, feasibility, and efficacy . Therefore, clarification of hypoxia-altered proteins together with follow-up functional studies are needed prior to clinical ASCs delivery to damaged tissues, particularly when stem cells from non-human donors are considered for testing.

Adipose tissue stem cells from livestock animals have gained increased attention in recent years due to their promising role in developing regenerative medicine protocols that can be utilized in veterinary medicine^22^ and humans. Of particular interest are large animals such as pigs that share multiple similarities with human and are necessary to conduct pre-clinical studies. To date, adult stem cells have been identified in a variety of pig tissues, including bone marrow^23^, endometrium^24^, ovary^25^, and abdominal fat^26^. However, only a limited number of studies have investigated porcine stem cells in the context of cutaneous wound healing^27^. Moreover, there are no data related to effect of hypoxia on pig ASCs proteome and the cell function.

The present study was undertaken to characterize pASCs from the subcutaneous fat of prepubertal domestic gilts. We evaluated the effect of hypoxia (1% O_2_) on pASCs proteome using two-dimensional difference gel electrophoresis (2D-DIGE) to quantify proteins and matrix-assisted laser desorption/ionization (MALDI) mass spectrometry to identify proteins with different abundances in hypoxia-treated pASCs. Finally, in vitro functional studies were performed to determine the impact of hypoxia on pASC properties associated with skin wound healing.

## Results

### Characterization of pASCs

**Figure 1:**
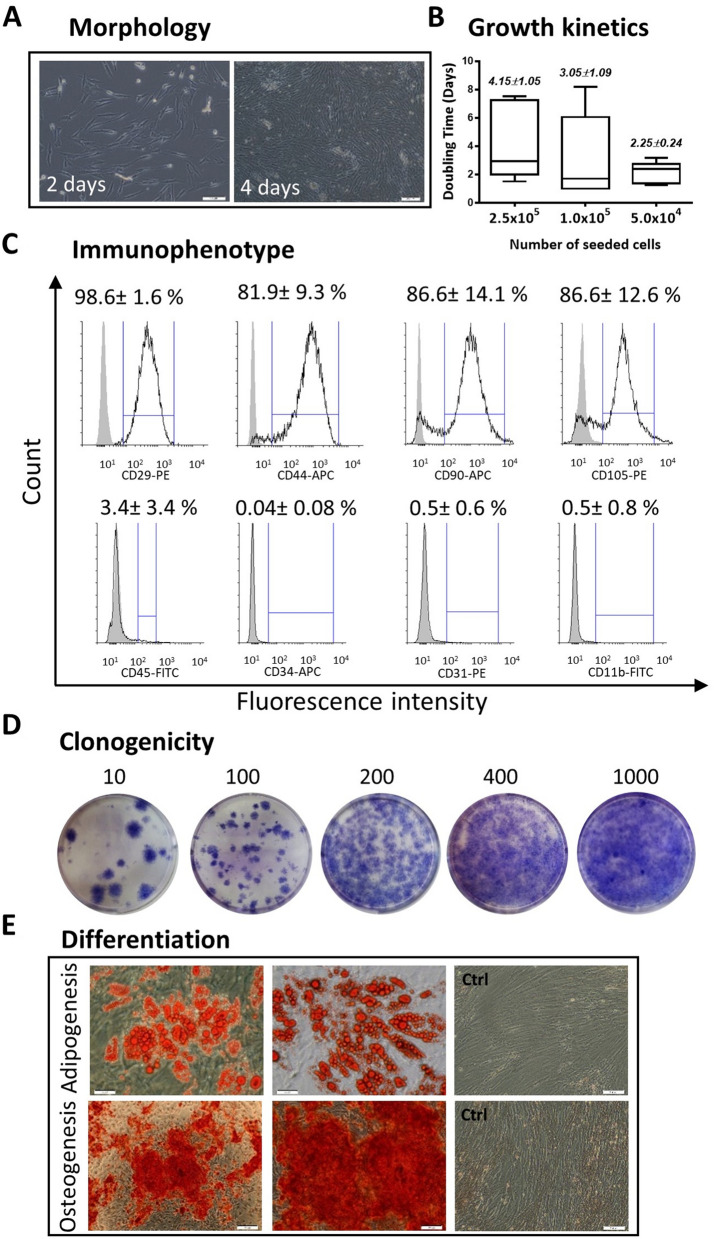
Characterization of pASCs. **(A**) Spindle-shaped morphology and rapid expansion at p = 0. **(B)** Growth kinetic demonstrating the influence of seeding density at 2.5 × 10^5^, 1.0 × 10^5^, or 5.0 × 10^4^ cells/well of 24-well plates (n = 9). **(C)** Flow cytometric analysis of surface markers exhibiting high expression of MSCs (CD29, CD44, CD90, CD105; black lines) and low or no presence of CD45, CD34, CD31, CD11b (n = 5–6). Gray lines indicate background fluorescence obtained with isotype control IgG. **(D)** Clonogenicity of pASCs reflected by the ability of single cells to form CFUs were seeded at densities of 10, 100, 200, 400, but not at 1000 cells/well of 6-well plates (n = 5). **(E)** pASCs induced for adipogenesis and osteogenesis and stained with Oil Red O and Alizarin Red, respectively (n = 4). Abbreviations: pASCs, pig adipose-derived stem cell; MSCs, mesenchymal stem cell. Scale bars: 200 µm.

Within 48 h after plating, pASCs were identified as plastic-adherent cells with spindle-like morphology (Fig. 1A). Because the cells showed a tendency toward rapid expansion (Fig. 1A), we examined the growth kinetics by seeding pASCs at densities of 5.0 × 10^4^, 1.0 × 10^5^, 2.5 × 10^5^ cells/well and assessed the DT at *p* = 1. However, estimations of cell doubling time (DT) revealed no effect of plating density on pASC growth dynamics (Fig. 1B, see Supplementary Table S1). Culture-expanded pASCs at passage 0 (*p* = 0) or passage 1 (*p* = 1) demonstrated several stem cell features. Immunophenotypically, pASCs showed a high percentage of cells with MSC markers including CD29 (98.6 ± 1.6%), CD44 (81.9 ± 9.3%), CD90 (86.6 ± 14.1%) and CD105 (86.6 ± 12.6%). The cells were negative for CD11b, CD31, and CD34; however, a small subpopulation of pASCs (3.4 ± 3.4%) showed expression of the hematopoietic marker CD45 (Fig. 1C). Clonogenicity assays revealed that pASCs plated at low concentrations (10, 100, or 200 cells/well) demonstrated the potential to form CFUs (Fig. 1D), while high-density plating (400 or 1000 cells/well) led to reduced clonogenicity regarding the extent of establishing of a cell monolayer (Fig. 1D). pASCs exposed to adipogenic or osteogenic differentiation-induction media displayed the capacity to differentiate into adipocytes or osteoblasts as evidenced by histological examination of lipid droplets stained with Oil Red O and calcium deposits stained with Alizarin Red S, respectively (Fig. 1E).

### Proteomic analysis of pASCs exposed to hypoxia (1% O_2_) or normoxia (21% O_2_)

The proteomic data showed that 93 protein spots, corresponding to 70 proteins, differed significantly (*p* < 0.05) between pASCs cultured for 24 h under hypoxia (1% O_2_) or normoxia (21% O_2_). Sixty two spots representing 42 proteins were enriched in cells exposed to low oxygen, whereas 31 spots corresponding to 28 proteins showed decreases upon hypoxic exposure (see Supplementary Table S2). Identified proteins were 1.03- 1.89-fold regulated by oxygen tension. The largest changes were observed in response to hypoxia for N-myc downstream regulated 1 (NDRG1) protein (fold change -1.89) and translocon-associated protein subunit delta isoform X2 (SSR4; fold change -1.78, see Supplementary Table S2). The group of 27 proteins corresponding to 31 spots that demonstrated fold-enrichment ≤ 1.10 were excluded from further analysis (see Supplementary Table S2 and Supplementary Figure S1).

**Figure 2:**
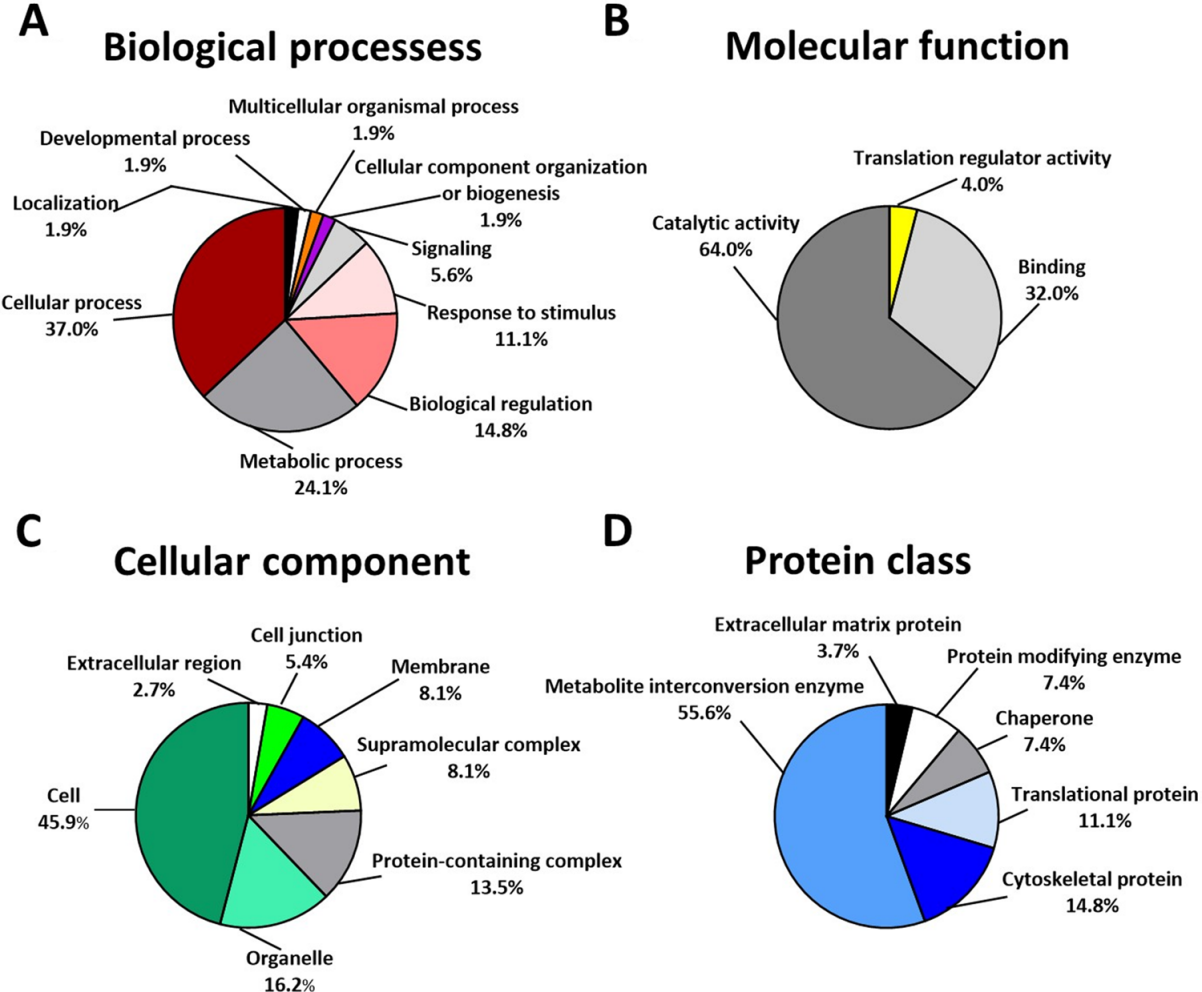
Classification of pASCs differentially expressed proteins upon 24 h exposure to hypoxia (1% O_2_) or normoxia (21% O_2_) according to GO classification. The pie charts illustrate differentially expressed proteins in terms of **(A)** biological processes, **(B)** molecular function, **(C)** cellular component, and **(D)** protein class. Abbreviation: GO, gene ontology.

To characterize functionality and define the types of proteins differentially expressed between pASCs treated with hypoxia or normoxia, their genes were classified into four main GO categories including “Biological processes,” “Molecular function,” “Cellular component,” and “Protein class” using the PANTHER Classification System (Fig. 2, distribution of hypoxia up- and down-regulated proteins between individual GO categories are presented in Supplementary Tables S3 and S4). Generally, with respect to “Biological processes,” proteins involved in cellular (37%) and metabolic (24.1%) processes comprised the largest group of proteins affected by hypoxia (Fig. 2A). Furthermore, according to the classification category of “Molecular function,” 64% of differentially expressed proteins identified by PANTHER Classification System demonstrated catalytic function, whereas 32.0% were found to be engaged in binding (Fig. 2B). In the “Cellular component” category, proteins associated with cell (45.9%) and organelle (16.2%) constituted the largest group of oxygen-sensitive molecules (Fig. 2C). Considering the “Protein class” cluster, many hypoxia-responsive proteins were represented by metabolite interconversion enzyme (55.6%) and cytoskeletal protein (14.8%) (Fig. 2D).

**Figure 3:**
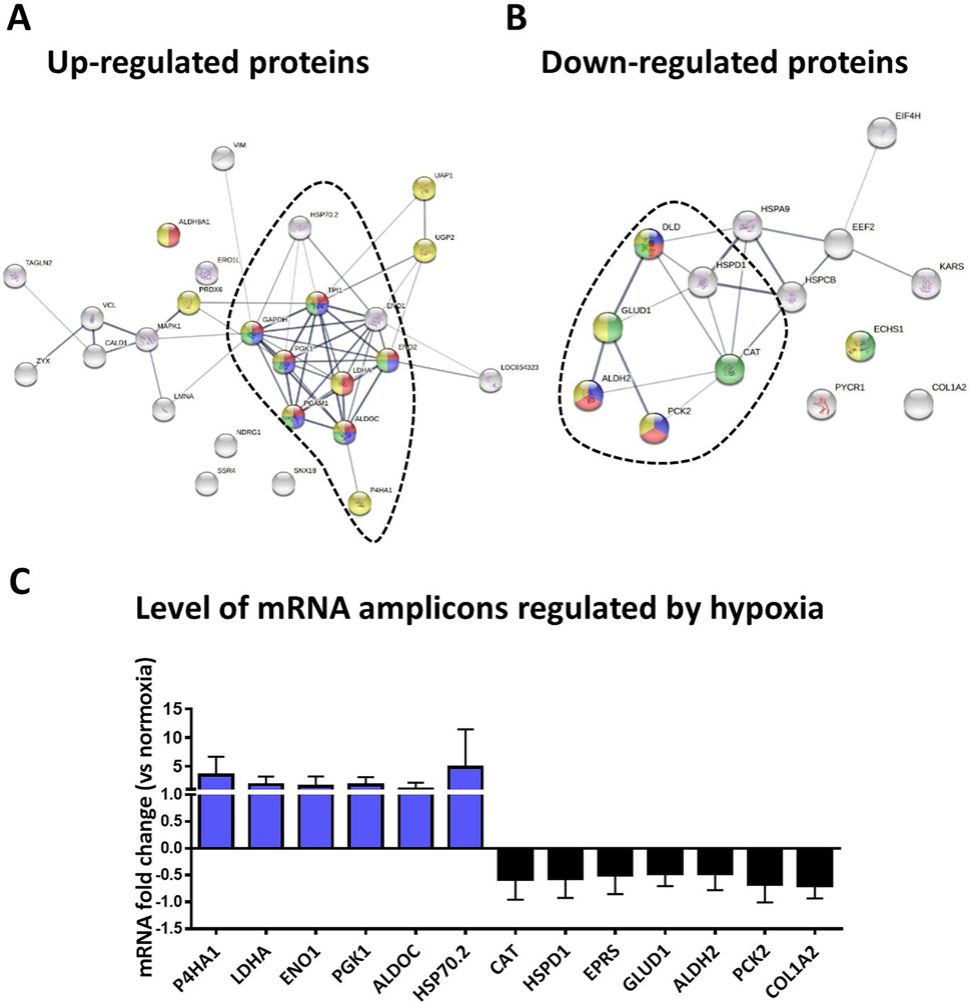
Protein–protein interaction networks for pASCs genes differentially expressed following hypoxic preconditioning and analyzed in the STRING online database, version 11.0. The line thickness indicates the strength of data (tight lines indicate high score, > 0.7; thin lines indicate medium score, > 0.4). **(A)** Interactions of the proteins up-regulated upon hypoxia. Red: proteins involved in glycolysis/gluconeogenesis. Blue: proteins involved in biosynthesis of amino acids. Green: proteins that participate in carbon metabolism. Yellow: proteins engaged in metabolic pathways. **(B)** Interactions of down-regulated proteins. Red: proteins involved in glycolysis/gluconeogenesis. Blue: proteins involved in puryvate metabolism. Green: proteins that participate in carbon metabolism. Yellow: proteins engaged in metabolic pathways. **(C)** The proteins with the strongest interactions in each group are marked by dotted lines (A, B) and were evaluated by real-time PCR. The differentially expressed proteins of pASCs treated in low and ambient oxygen concentrations are shown in Tables 1 and 2.

STRING database query (Fig. 3) and subsequent KEGG pathway analysis allowed us to specify processes enriched by differentially abundant proteins. Of the 27 proteins up-regulated upon hypoxia, 26 were identified in the STRING database (Fig. 3A). The proteins that interacted with each other (52 edges) and demonstrated the strongest connections (average local clustering coefficient, 0.598; protein–protein interaction [PPI] enrichment *p* value < 1.0e−16) are involved in glycolysis/gluconeogenesis, biosynthesis of amino acids, and carbon metabolism (Fig. 3A; Table 1, see Supplementary Table S3).

**Table 1:** Pathways enriched by proteins up-regulated in hypoxia (1% oxygen).

KEGG Pathway	Genes
Glycolysis / Gluconeogenesis	ALDH9A1, ALDOC, ENO2, GAPDH, LDHA, PGAM1, PGK1, TPI1
Biosynthesis of amino acids	ALDOC, ENO2, GAPDH, PGAM1, PGK1, TPI1
Carbon metabolism	ALDOC, ENO2, GAPDH, PGAM1, PGK1, TPI1
Focal adhesion	MAPK1, VCL, ZYX
Adherens junction	VCL MAPK1
Arginine and proline metabolism	P4HA1
Metabolic pathways	ALDH9A1, ALDOC, ENO2, GAPDH, LDHA, P4HA1, PGAM1, PGK1
Pyruvate metabolism	ALDH9A1, LDHA
Central carbon metabolism in cancer	MAPK1, PGAM1
Glucagon signaling pathway	LDHA, PGAM1
HIF-1 signaling pathway	ENO2, GAPDH, MAPK1
Fructose and mannose metabolism	ALDOC, TPI1
Protein processing in endoplasmic reticulum	ERO1L, HSP70.2, SSR4
Amino sugar and nucleotide sugar metabolism	UAP1, UGP2

Importantly, the majority of hypoxia up-regulated proteins (ALDOC, TPI1, GAPDH, PGK1, PGAM1, ENO1, ENO2, LDHA) were linked with anaerobic metabolism (glycolysis). The second large group of molecules that increased with hypoxia comprised proteins involved in focal adhesion and intracellular communication (MAPK1, ZYX, VCL). With regard to proteins associated with wound healing, we found that prolyl 4-hydroxylase subunit alpha-1 precursor (P4HA1) was increased with hypoxia (Fig. 3A). In addition, heat shock protein 70.2 (HSP70.2) exhibited increased abundance in low oxygen environment (Fig. 3A).

In the group of hypoxia-down-regulated proteins, there were 14 that were included in the protein network. The most connected proteins (average local clustering coefficient, 0.4; PPI enrichment *p* value < 5.e−10) participate in glycolysis/gluconeogenesis, tryptophan, pyruvate and carbon metabolism, and biosynthesis of amino acids, as well as degradation of valine, leucine, and isoleucine (Fig. 3B, Table 2, see Supplementary Table S4).

**Table 2:** Pathways enriched by proteins down-regulated in hypoxia (1% oxygen).

KEGG Pathway	Genes
Glycolysis/Gluconeogenesis	ALDH2, DLD, PCK2
Tryptophan metabolism	ALDH2, CAT, ECHS1
Pyruvate metabolism	ALDH2, DLD, PCK2
Carbon metabolism	CAT, DLD, ECHS1, GLUD1
Valine, leucine and isoleucine degradation	ALDH2, DLD, ECHS1
beta-Alanine metabolism	ALDH2, ECHS1
Fatty acid degradation	ALDH2, ECHS1
Metabolic pathways	ALDH2, DLD, ECHS1, GLUD1PCK2
Lysine degradation	LDH2, ECHS1
RNA degradation	HSPA9, HSPD1
Proximal tubule bicarbonate reclamation	GLUD1, PCK2
Glyoxylate and dicarboxylate metabolism	CAT, DLD
Necroptosis	GLUD1, HSPCB (HSP90AB1)
Citrate cycle (TCA cycle)	DLD, PCK2
Propanoate metabolism	DLD, ECHS1
PI3K-Akt signaling pathway	COL1A2, HSPCB (HSP90AB1), PCK2
AMPK signaling pathway	EEF2, PCK2
FoxO signaling pathway	CAT, PCK2
Tuberculosis	HSPA9, HSPD1

Moreover, down-regulation of phosphoenolpyruvate carboxykinase [GTP] (PCK2) and dihydrolipoyl dehydrogenase (DLD), both of which play roles in the citrate cycle, confirmed that aerobic metabolism is suppressed in hypoxia-treated cells. With regard to proteins critical/fundamental for cutaneous healing, collagen type I chain alpha 2 (COL1A2) was down-regulated following hypoxia (Fig. 3B. Table 2). Among the hypoxia-down-regulated proteins, we found heat shock proteins including HSPCB (also known as HSP90AB1), HSPD1, and HSPA9 (Fig. 3B). mRNA level verification of selected hypoxia-responsive proteins confirmed their up- or down-regulation under low oxygen (Fig. 3C).

### Effect of hypoxia on pASCs functional features

**Figure 4:**
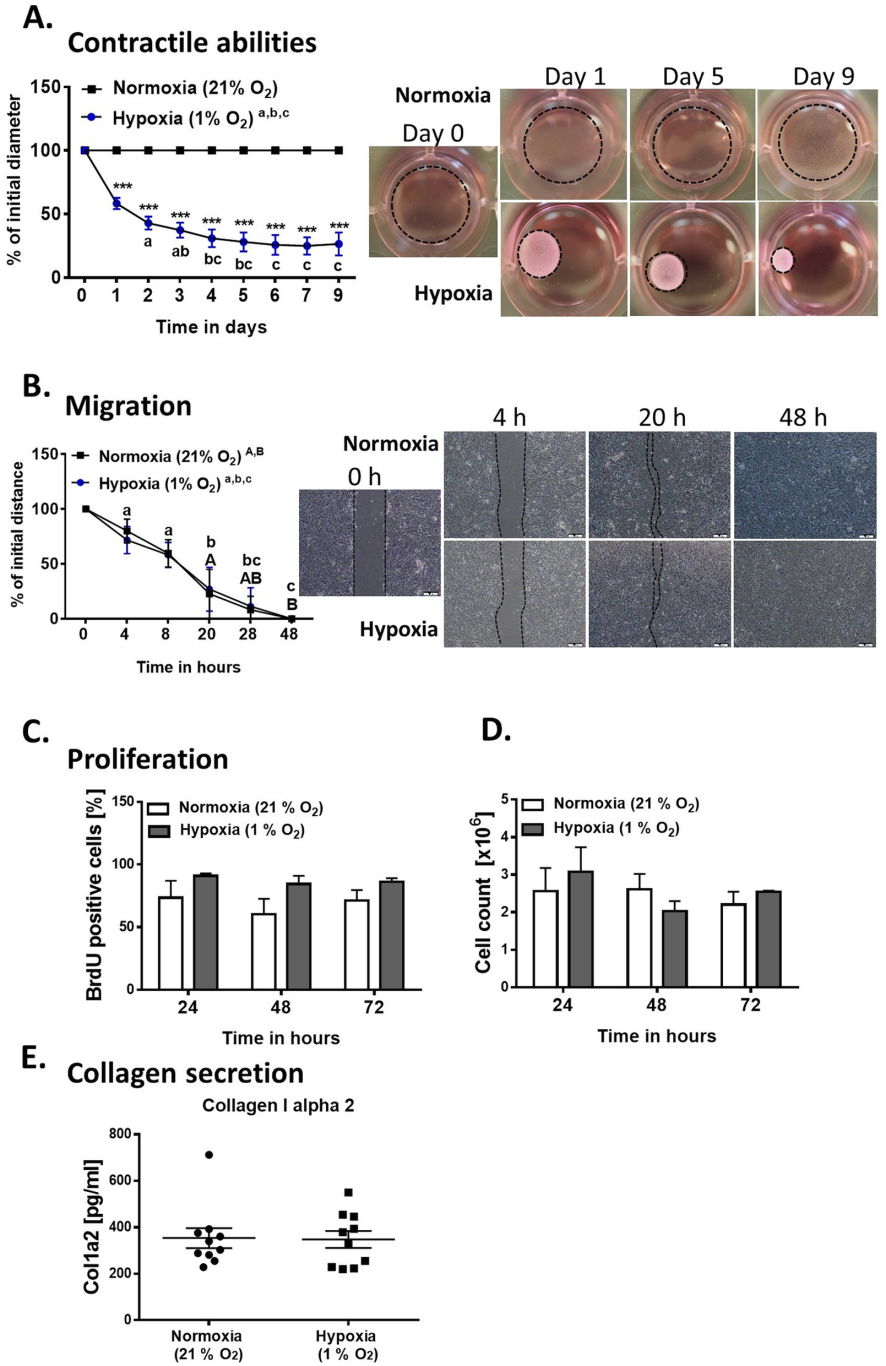
Functional examination of pASCs exposed to 24 h of hypoxia (1% O_2_) or normoxia (21% O_2_). **(A)** Development of pASCs contractile abilities during 9 days of culture in an oxygen-deficient environment (*p* < 0.001; n = 4–5). Representative images show the contractile abilities of pASCs at days 0, 1, 5, 9 of culture under normoxia and hypoxia. **(B)** Wound healing (scratch) in vitro assay demonstrated no effect of hypoxia on pASCs migratory abilities (n = 8). Hypoxic preconditioning had no effect on pASC proliferation assessed by **(C)** BrdU incorporation (n = 4) and **(D)** cell count (n = 4). **(E)** Low oxygen content did not impact the level of collagen type 1 alpha 2 secreted to the culture medium (n = 10). Mean ± SD. ***p < 0.001. Panel **(A)** Asterisks indicate significant differences between pASCs exposed to hypoxia and normoxia. Small letters (a, b, c) show significant differences (*p* < 0.001) of pASC (**A**) contractile and (**B**) migratory abilities following hypoxia relative to day 0 and between consecutive experimental days. Big letters (A, B) indicate significant differences (*p* < 0.001) of pASC (**B**) migratory abilities in normoxia relative to day 0 and between consecutive experimental days.

We performed a series of in vitro studies to define the role of hypoxia on pASCs functional features, particularly those involved in wound healing. Collagen gel contraction assays were performed to assess pASC contractile abilities that are crucial for wound closure in vivo. Hypoxia stimulated pASC-mediated contraction of collagen gels over time relative to cells exposed to normoxia (*p* < 0.001 for days 1–9, Fig. 4A; see Supplementary Table S5a). Low oxygen gradually promoted pASC contractile abilities between days 0 and 9 with gel size reductions of 61.22 ± 9.88%, 51.52 ± 9.58%, 42.45 ± 8.88%, 36.51 ± 10.39%, 29.81 ± 12.26%, 25.91 ± 13.75%, 23.47 ± 14.32%, 23.23 ± 12.39 and 24.45 ± 16.22% at days 1, 2, 3, 4, 5, 6, 7 and 9, respectively (*p* < 0.001 for each day; Fig. 4A; see Supplementary Table S5b.). Interestingly, pASCs cultured under ambient oxygen concentration showed no contractile potential (Fig. 4A; see Supplementary Table S5b.). Migratory properties of pASCs were examined with scratch assays. Low oxygen exposure had no effect on motility compared to atmospheric oxygen level (Fig. 4B; see Supplementary Table S6a). Based on the measurements performed at 4, 8, 20, 28, and 48 h (all *p* < 0.001 in the hypoxia group) after scratching, gradual closure was observed for both culture conditions (Fig. 4B; see Supplementary Table S6b). Because pASCs were pre-treated with mitomycin C, the “wound closure” capacity was exclusively due to cell migration. However, considering that wound healing is also driven by cell proliferation we examined pASC propagation abilities using BrdU incorporation and cell counting. Neither approach showed any effect of hypoxia on pASC proliferation at 24, 48, or72 h compared to normoxic cells (Fig. 4C, D; see Supplementary Tables S7 and S8).

Furthermore, given that collagen deposition is an important hallmark of cutaneous healing which is consistent with our proteomic data (see Fig. 3B. and Table 2), we next examined the impact of hypoxia on pASCs ability to produce collagen type I alpha 2 (Fig. 4E). The results of these analyses showed that there was no change in collagen secretion to the culture media after 24 h of hypoxia (Fig. 4E; see Supplementary Table S9a and S9b).

## Discussion

There has been tremendous progress in pre-clinical studies of human ASCs in the context of their use in regenerative medicine. Although considerable advancement in identification and characterization of animal ASCs has been observed, these cells have not received the attention paid to their human equivalents, so animal-derived ASCs are a relatively new area to explore. The present studies provide a protocol of efficient ASCs isolation from the subcutaneous fat of domestic pigs (pASCs) and their culture-expanded characterization.

For optimal pASCs recovery, we adapted a method originally established to isolate human ASCs^4,19^. Because no criteria have been established to identify porcine stem cells, we facilitated those defined by The International Federation for Adipose Therapeutics and Science (IFATS) and the International Society for Cellular Therapy (ISCT) to identify human ASCs^28^. These criteria were also used in other studies for characterization of pig ASCs derived from visceral^29^ and subcutaneous interscapular sites and buccal fat pads^30^. Our results show that pASCs isolated from the subcutaneous fat of prepubertal domestic gilts fulfill the criteria for MSCs with regard to morphology, clonogenicity, differentiation potential and immunophenotype. Flow cytometry analysis confirmed the expression of primary MSC surface markers (CD29, CD44, CD90, CD105) that is with agreement with the studies on human^31^ and pig ASCs^32^. Likewise, the presence of small fraction of CD45 positive cells has been demonstrated in numerous independent investigations^33–35^.

The major goal of the present study was to examine the effect of hypoxia on the pASCs protein signature with particular attention to proteins associated with wound healing. Because damaged tissues are often characterized by oxygen deprivation, much work has been done to identify strategies that improve ASCs survival in hypoxic environments^15,36^. In this respect, hypoxia pre-conditioning plays a protective role in human ASCs^16^ and concomitantly enhances their reparative/regenerative properties^15^.

In our study, we confirmed the observation of Riis *et al.*^21^ that hypoxic preconditioning of human ASCs caused subtle differences in protein expression. Changes in protein abundance ranged between 1.89 and 1.03 in our study. The largest changes were observed in response to hypoxia for N-myc downstream regulated 1 (NDRG1) protein (fold change -1.89, see Supplementary Table S1). Similarly to our study, Riis *et al.* showed that differences in protein levels between human ASCs cultured in hypoxic and normoxic conditions were less than two-fold for most identified proteins^21^. Interestingly, mass spectrometry analysis of the cells revealed that a relatively small fraction of the proteome (9.6%) is significantly affected by hypoxia^21^, which is comparable to the 9.16% of affected proteins in our study.

A variety of proteins were differentially expressed between hypoxia- and normoxia-exposed cells. However, only a small proportion of these were strictly related to the process of cutaneous wound healing. In particular, low oxygen increased the expression of P4HA1, which catalyzes the formation of 4-hydroxyproline that is essential for the correct folding of triple helical collagen molecules^37^. This is consistent with research on human ASCs that showed increased P4HA1 and P4HA2 levels following 24 h of hypoxia^21^. The authors also demonstrated that hypoxia stimulated the expression of other proteins involved in matrix regulation including COL1A1, COL3A1, and COL7A1^21^. Surprisingly, in our study we found that although hypoxia increased the levels of P4HA1, it concomitantly led to decreased expression. These findings are in agreement with a report by Frazier *et al.*^19^ that evidenced lower levels of COL1A1, COL1A2, and fibronectin released to CM upon hypoxia exposure of human ASCs. Since overabundance of these proteins were found in multiple models of tissue fibrosis including skin^38^, the hypoxia-induced decrease might suggest that hypoxia pre-conditioning prevents ASCs from excessive ECM deposition.

Consistent with the literature, exposure to low oxygen causes cells to switch from oxidative phosphorylation to glycolysis, which becomes the primary method for ATP production^21,39,40^. As expected, we found that the largest group of proteins affected by hypoxia were enzymes in the glycolytic pathway including ALDOC, TPI1, GAPDH, PGK1, PGAM1, ENO1, ENO2, and LDHA. These metabolic alterations provide the energy necessary for cell adaptive responses and thus support cell survival^16^ and function^41,42^.

Hypoxia is a natural shock signal to cells, so we put our focus on stress-related proteins. Oxygen deficiency increased the presence of heat shock 70 kDa protein 1B (HSP70.2) in pASCs. HSP70 is a crucial negative regulator of the mitochondrial pathway of apoptosis that is able to block cell death at both pre- and post-mitochondrial levels^43^. Recent studies demonstrated that HSP70 upregulation in induced pluripotent stem cells-derived cardiomyocytes (iPS-CMs) exposed to hypoxia promoted cell survival by suppressing apoptotic pathways^44^. Clinically, this observation implies that increased HSP70 might improve the survival of transplanted stem cell and enhance their therapeutic potential. It was previously shown that high HSP70 levels correlate with proper wound healing, likely by enhancing endothelial cell function, whereas its weak or lack of expression results in chronic wound development^45^. In addition, delivery of RAW264 macrophages pretreated with HSP70 to full-thickness cutaneous wounds in mice accelerated wound closure by stimulating macrophage‐mediated phagocytosis of wound debris^46^. Together, these data suggest that cells subjected to hypoxia release specific pro-repair and/or pro-regenerative agents and might be adopted as a “carrier cell system” for delivering molecules that enhance damaged tissue recovery.

The initial expectation was that low oxygen tension may increase the expression of all identified HSPs, as in general they play an important protective role in the cellular response to hypoxia. However, proteomic data revealed that the presence of some of HSPs representatives, such as stress-70 protein, mitochondrial (HSPA9), heat shock 90kD protein 1 beta (HSP90AB1), and mitochondrial 60 kDa heat shock protein (HSPD1), were decreased following hypoxia. Among them, HSP90 exhibited well-documented abilities to maintain stem cell fate^46^. Indeed, the results showed that HSP90 is essential for maintaining pluripotency of mouse embryonic stem cells (ESCs) through regulating the pluripotency factors Oct4 and Nanog. Dramatic decreases in the expression levels of both HSP90α and HSP90β were observed during ESCs differentiation into embryoid bodies. Furthermore, HSP90 inhibition led to lower *Oct4* mRNA levels and increased markers for mesoderm lineage^47^. Along this line, the decrease in some HSPs following hypoxia exposure might indicate a reduced stress defense capacity of pASCs and/or an ongoing differentiation process. Although these concepts remain to be elucidated, the increase in pASCs contractility led us to presume that hypoxia improves acquisition of the smooth muscle cell phenotype (See Supplemental Fig. S2). This viewpoint is consistent with the results obtained from human ASCs, showing that low oxygen conditions influenced their differentiation into smooth muscle cells^17^.

With regard to wound healing, the effect exerted by HSP90 remains under debate as the protein has been shown to improve skin cell motility and accelerate wound closure^48^, whereas others reported that overexpression of HSP90 led to excessive collagen synthesis and keloid formation^49^. Altogether, the consequences of decreased HSP90AB1, HSPD1, and HSPA9 in response to hypoxia require further investigations.

The cellular response to hypoxia involved cell components associated with focal adhesion and cell to cell communication. Besides regulating cell morphology and polarity, these structural systems also control cell dynamics essential for multiple physiological processes such as migration, adhesion, ECM remodeling, and signaling^50–52^. The present study revealed that hypoxia increased pASC expression of zyxin (ZYX), vinculin (VCL), vimentin (VIM), and caldesmon 1 (CALD1). These results are in agreement with data obtained from human ASCs demonstrating increased integrin alpha 5 (ITGA5) and intercellular adhesion molecule 1 (ICAM1) in response to hypoxia^21^. The increase in proteins responsible for intracellular communication and cells’ connections with the extracellular environment suggest that hypoxia-exposed pASCs might exert their effect in injured tissue by replenishing damaged cells. However, this concept merits further studies of pASCs ability to differentiate into the phenotype of injured tissue (e.g., skin) and further in vivo evaluation.

While oxygen deprivation did not affect cell migration, proliferation, or collagen type 1 alpha 2 secretion, hypoxia considerably enhanced pASCs contractile abilities. These findings are consistent with those reported by Wang *et al.* that demonstrated increased contractility of human ASCs cultured at 5% oxygen^17^. Other investigators have reported that stimulation of processed lipoaspirate cells with the cholinergic agonist carbachol or 60 mM KCl into smooth muscle-like cells differentiated from human ASCs induced their contractile function in standard ambient culture conditions (20% O_2_)^53,54^. The lack of extrinsic stimuli in culture medium might partially explain the static behavior of pASCs exposed to normoxia. This topic requires further investigation. There is no definitive answer for the mechanisms that regulate pASCs functions in vitro, and further assessments are needed.

To the best of our knowledge, this is the first report demonstrating the effect on hypoxia on the proteome and functional characteristics of pASCs. In veterinary medicine, pASCs xenotransplantation can be an alternative to costly and inconvenient autotransplantation. Currently, ASC-related therapies are largely used to treat musculoskeletal tissue injury in horses^55^ and dogs^56^. This trend advocates for the need of animal ASC availability as an off-the-shelf product that will allow vets to use them directly at the point of care. With regard to humans, since wound healing studies have been limited by a lack of relevant in vivo experimental models studies, autotransplanting pig stem cells into damaged tissues can provide a clinically relevant model to evaluate the outcome of allogenic cell grafts. Furthermore, for a number or reasons including size, anatomical, and physiological similarities with humans, the pig is a preferred donor species. The results of preclinical studies of transplantation of pig cells (e.g., islets, neuronal cells, or hepatocytes) into non-human primates have encouraged their further use in clinical trials^57^. Given that significant progress has been made in many areas of xenotransplantation, it is likely that pASCs will be considered for cell-based therapies in humans. On the other hand, the wide availability of human ASCs and their well-documented therapeutic properties support the consensus that future application of ASCs in human medicine will be conducted mostly in an autologous form.

## Conclusion

In searching for alternative sources of stem cells to facilitate wound healing in animals and/or humans, we isolated and characterized of ASCs from the subcutaneous fat of domestic pigs and showed that they fulfilled the criteria of MSCs. The data demonstrated that hypoxia (1% O_2_) altered the expression of proteins associated with cell metabolism, ECM components, and intracellular communication. Changes induced by oxygen deficiency might suggest pASCs potential to improve wound healing by preventing excessive collagen deposition and enhancing cell-to-cell communication and ECM interaction. By combining a proteomics approach with functional pASC assessment following hypoxia, we performed the initial step toward qualifying pASCs as attractive candidates in cell-based applications for wound healing therapeutics. However, as present report characterizes pASCs exclusively in the in vitro settings further in vivo studies on animal models of wound healing are required to verify pASCs contribution in this process. Moreover, given that human ASCs exert their effects on wound healing in a paracrine manner, further study of the pASC secretome is necessary to fully understand their specificity. This will require additional studies exploring the mechanism underlying pASC action in injured skin and further pre-clinical evaluation in both rodents and large animal models.

## Materials and methods

### Isolation of pASCs

Subcutaneous fat samples collected from back and leg ham regions of each donor were obtained post-mortem from domestic gilts (*Sus scrofa;* n = 10) in a slaughterhouse. pASCs were isolated according to the method describe by Frazier *et al.*^19^, with modifications. Briefly, the adipose tissue was washed with phosphate-buffered saline (PBS), 70% ethanol, and 10% betadine; minced with scissors; and enzymatically digested with 0.1% collagenase type I (Sigma-Aldrich Co.) in PBS with 100 IU/mL penicillin and 100 μg/mL streptomycin (Sigma-Aldrich Co.) for 3 h at 37 °C. The floating adipocytes were separated from the stromal vascular fraction (SVF) by centrifugation (250×*g*) for 5 min at 28 °C. The supernatant containing mature adipocytes was discarded, and the remaining pellet identified as the SVF was suspended in Dulbecco’s minimum essential medium (DMEM)/F12 (Sigma-Aldrich Co.) supplemented with 10% fetal bovine serum (FBS; Gibco by Thermo Fisher Scientific), 1% penicillin/streptomycin, and 0.25 µg amphotericin B (Sigma-Aldrich Co.). The cells were seeded in T75 cell culture flasks at 37 °C in a 5% CO_2_ and 95% air atmosphere (*p* = 0). After 48 h, nonadherent and dead cells were removed, and fresh Adipose-Derived Stem Cell Basal Medium (ADSC-BM; Poietics, Lonza) supplemented with 10% FBS, L-glutamine, gentamicin/amphotericin B (ADSC-GM SingleQuots; Poietics Lonza) was added. The primary cultured pASC (*p* = 0) were cryopreserved in cryopreservation medium (10% dimethyl sulfoxide [DMSO], 10% DMEM/F12, 80% FBS) and frozen in liquid nitrogen before thawing for individual assays.

### Flow cytometry analysis of pASC surface markers

Analysis of the pASC (n = 5–6) phenotypic profile by flow cytometry was conducted by resuspending cells in PBS at a concentration of 0.5 × 10^6^ per tube and staining for 30 min on ice with the following antibodies: CD11b-FITC (Exbio Praha, a.s.), CD29-PE (Exbio Praha, a.s.), CD31-PE (Exbio Praha, a.s.), CD34-APC (Exbio Praha, a.s.), CD44-APC (Exbio Praha, a.s.), CD45-FITC (Bio-Rad Laboratories Inc.), CD90-APC (BD Pharmingen), and CD105-PE (Exbio Praha, a.s.). Isotype-matched immunoglobulins IgG1-FITC (Exbio Praha, a.s.) and IgG2-PE (Exbio Praha, a.s.) served as controls for nonspecific immunofluorescence. The labeled cells were analyzed using a BD LSRFortessa Cell Analyzer flow cytometer (Becton Dickinson) and BD FACSDiva v6.2 Software (Becton Dickinson). Histograms were overlapped using Flowing Software Version 2.5.0 (Cell Imaging Core, Turku BioImaging; Turku Centre for Biotechnology, University of Turku, Ăbo Akademi University). The total number of cells analyzed for each sample was 10,000.

### Clonogenicity

To assess pASC ability to form colony-forming units (CFUs), cells (n = 5) were seeded in 6-well plates at a clonal density of 10, 100, 200, 400, and 1000 cells per well. The cells were cultured at 37 °C with 5% humidified CO_2_ for 21 days in ADSC-BM supplemented with 10% FBS, L-glutamine, and gentamicin/amphotericin. Fresh medium was replaced every 3–4 days. Clones were fixed with 4% formaldehyde and stained with Giemsa (Sigma-Aldrich Co.).

### Differentiation

pASCs (n = 4) were seeded in duplicate at the concentration of 5.0 × 10^4^ into 24-well plates (*p* = 1) and cultured until they reached 60–70% confluency. Adipogenic differentiation was induced according to the procedure described previously by Yu *et al.*^58^. Briefly, adipogenic medium I consisted of DMEM/F12 supplemented with 5% FBS, 66 μM biotin (Sigma-Aldrich Co.), 34 μM d-pantothenate (Sigma-Aldrich Co.), 200 nM insulin (Sigma-Aldrich Co.), 1 μM dexamethasone (Sigma-Aldrich Co.), 250 μM isobutyl-methylxanthine (IBMX, Sigma-Aldrich Co.), and 5 μM troglitazone (Sigma-Aldrich Co.). Adipogenic medium II consisted of DMEM/F12 supplemented with 5% FBS, 66 μM biotin, 34 μM d-pantothenate, 200 nM insulin, and 1 μM dexamethasone. The pASCs were incubated for 3 days in adipogenic medium I, and for the next 11 days, cells were cultured in adipogenic medium II at 39 °C in 21% O_2_ and 5% humidified CO_2_. For osteogenic induction, Stem-Pro1 Osteogenesis Differentiation Kits (Life Technologies by Thermo Fisher Scientific) were used according to the manufacturer’s instructions.

### Growth kinetics

pASCs (n = 9) were seeded in duplicate in 24-well plates at densities of 5.0 × 10^4^, 1.0 × 10^5^, and 2.5 × 10^5^cells/well in ADSC-BM supplemented with 10% FBS, L-glutamine, and gentamicin/amphotericin. At 80–90% confluence, cells were passaged by digestion with 0.05% trypsin, counted, and reseeded. Serial passaging was continued until the harvested cell numbers dropped below the initially plated number. Cell-doubling times (DT) were calculated by using the following formula: DT = CT/ln(Nf/Ni)/ln(2), where DT is the cell-doubling time, CT the cell culture time, *N*f is the final number of cells, and *N*i is the initial number of cells^59^.

### pASC hypoxia culturing

pASCs (*p* = 1; n = 4) were plated at a density of 1.5 × 10^5^ cells/cm^2^ in ADSC-BM medium supplemented with 10% FBS, L-glutamine, and gentamicin/amphotericin. At 80–90% confluence, cells were washed with PBS, and medium was replaced with serum-free ADSC-BM with L-glutamine. For normoxic cultures, pASCs were cultured at 21% O_2_ and 5% CO_2_. For hypoxia studies, pASCs were maintained in a multigas incubator (Panasonic MCO-5M-PE) supplied with a gas mixture composed of 1% O_2_, 5% CO_2_, and balanced nitrogen for the duration of the experiment (24 h). pASCs isolated from the same donors (n = 4) were used for study both oxygen conditions.

### Proteomics

#### Sample preparation

pASCs cultured for 24 h in hypoxia (n = 4) or normoxia (n = 4) were collected in DIGE labeling buffer (30 mM Tris, 7 M urea, 2 M thiourea, and 4% CHAPS), sonicated for 15 s VC-13 PB (Sonic), set at 35% relative output, and centrifuged (10,000×*g* for 15 min, at 4 °C). Supernatants were precipitated using the 2-D Clean-up Kit (GE Healthcare), and pellets were re-suspended in DIGE buffer. The protein concentration was measured by the method of Bradford^60^, using a Coomassie Plus Kit (Thermo Scientific) with bovine serum albumin as the standard.

#### Fluorescence labeling of pASCs proteins with CyDyes and 2D-DIGE, Image Acquisition and Quantitative Analysis, MALDI TOF/TOF protein identification

Details are provided in Supplementary Information Methods and Supplementary Table S10.

### Gene ontology (GO) annotation and bioinformatics analyses

The GI accession numbers of identified proteins were mapped to the UniProtKB database (www.uniprot.org), and *Sus scrofa* was selected as the organism. The gene ontology (GO) annotation of differentiated proteins was performed by using online bioinformatics tools of PANTHER Classification System, version 14.1 (released 2019-04; https://pantherdb.org/). The proteins that differed in abundance between hypoxia- and normoxia- treated pASCs were classified based on the following GO categories: “biological processes,” molecular function,” “cellular component,” and “protein class.” The Kyoto Encyclopedia of Genes and Genomes (KEGG) was used for an annotation of biological pathways involved in the cellular response to hypoxia^60–63^. The analysis of potential protein–protein interactions were performed with the Search Tool for the Retrieval of Interacting Genes (STRING) online database, version 11.0 (https ://string-db.org)^64^ with a medium confidence score cutoff of 0.4.

### Validation of proteomic results by real-time polymerase chain reaction (PCR)

Details are provided in Supplementary Information Methods and Supplementary Table S11.

### Collagen gel contraction assay

Three-dimensional collagen gels were prepared according to a previously described protocol^65^. Details are provided in Supporting Information Methods.

### In vitro wound migration assay

Scratch assays were performed using a previously described procedure^66^. Details are provided in Supporting Information Methods.

### Proliferation assay

Proliferation of pASC (*p* = 1) was assessed by incorporation of 5′-bromo-2′-deoxyuridine (BrdU, BD Pharmingen) and by counting using automatic cell counter (Life Technologies by Thermo Fisher Scientific). Cells (n = 4) were plated on 60-mm Petri dishes at a density of 2.0 × 10^6^ cells and cultured in ADSC-BM with 10% FBS, L-glutamine, and antibiotics. At 50–60% confluence, the culture medium was replaced, and plates were continually cultured at 37 °C under normoxia (21% O_2_, 5% humidified CO_2_) or hypoxia (1%, O_2_, 5% humidified CO_2_) conditions for 24, 48, and 72 h. The cells were then washed with PBS, and BrdU was added at 10 μM for 16 h. Subsequently, pASCs were detached with 0.025% trypsin with EDTA and counted. Then, the cells were fixed, treated with DNase to expose BrdU epitopes, and labeled with FITC-conjugated anti-BrdU antibodies according to manufacturer’s instruction (FITC BrdU Flow Kit BD Pharmingen). Flow cytometry assays were performed using a BD LSRFortessa Cell Analyzer flow cytometer (Becton Dickinson) and BD FACSDiva v6.2 Software (Becton Dickinson). The total number of cells analyzed for each sample was 20,000.

### Enzyme-linked immunosorbent assays for Col1a2

The concentrations of collagen type 1α2 in conditioned media (CM) collected from pASCs (n = 10) cultured under hypoxia or normoxia were determined using Col1α2 enzyme immunoassay kits (Cloud-Clone Corp.) according to the manufacturer’s protocol. The standard curve for Col1a2 ranged from 3.12 to 2000 pg/mL. The intra- and inter-assay coefficients of variation (CV) were < 10% and < 12%, respectively.

### Statistical analysis

In order to conduct statistical analyses of pASCs growth kinetics (Fig. 1B), contractile abilities (Fig. 4A), migration (Fig. 4B), proliferation (Fig. 4C, D) and collagen I alpha 2 secretion (Fig. 4E) linear mixed-effects models were used, in which individuals were included as random intercept and different factors and their interactions were included as fixed effects. In case of insignificant random effects, linear models were used instead. As long as models were valid, based on them lsmeans were calculated and compared (for mixed-effects models Kenward-Roger method for degrees of freedom was used; Tukey p-value adjustment used where appropriate). All calculations were performed in R (ver. 3.5.3) using packages: lme4 (ver. 1.1-21), lmerTest (ver. 3.1-0), emmeans (ver. 1.4.1) and tidyverse (ver. 1.2.1). Data are expressed as mean ± standard deviation. Graphs (Figs. 1B, 2A–D, 3C, 4A–D) were performed using GraphPad PRISM, version 6.02 software (GraphPad Software). Proteomic data were analysed using paired t-test in biological variation analysis module of DeCyder Differential Analysis Software (5.02 software, GE Healthcare; Supplementary Table S2). All spots with p-value smaller than 0.05 were considered as differentially expressed.

### Ethics approval

The study protocol was approved by the Local Ethics Committee for Experiments on Animals, University of Warmia and Mazury, Olsztyn, Poland (Agreement No. 67/2018). The study was carried out in accordance with European Union Directive 2010/63/EU (OJEU, 2010. Official Journal of the European Union. Directive 2010/63/EU of the European Parliament and of the Council on the Protection of Animals Used for Scientific Purposes).

## Supplementary information

Supplementary information.

## Data Availability

The data that support the findings of this study are available from the corresponding author JB upon reasonable request.

## References

[1] Kolle SF (2013). Enrichment of autologous fat grafts with ex-vivo expanded adipose tissue-derived stem cells for graft survival: a randomised placebo-controlled trial. Lancet.

[2] Lee HC (2012). Safety and effect of adipose tissue-derived stem cell implantation in patients with critical limb ischemia: a pilot study. Circ. J. Off. J. Jpn. Circ. Soc..

[3] Thesleff T (2011). Cranioplasty with adipose-derived stem cells and biomaterial: a novel method for cranial reconstruction. Neurosurgery.

[4] Bunnell BA, Flaat M, Gagliardi C, Patel B, Ripoll C (2008). Adipose-derived stem cells: isolation, expansion and differentiation. Methods.

[5] Gimble JM, Katz AJ, Bunnell BA (2007). Adipose-derived stem cells for regenerative medicine. Circ. Res..

[6] Kuo YR (2016). Adipose-derived stem cells accelerate diabetic wound healing through the induction of autocrine and paracrine effects. Cell Transplant..

[7] Nie C (2011). Locally administered adipose-derived stem cells accelerate wound healing through differentiation and vasculogenesis. Cell Transplant..

[8] Strong AL (2015). Characterization of a murine pressure ulcer model to assess efficacy of adipose-derived stromal cells. Plast. Reconstr. Surg. Glob.Open.

[9] Ebrahimian TG (2009). Cell therapy based on adipose tissue-derived stromal cells promotes physiological and pathological wound healing. Arterioscler. Thromb. Vasc. Biol..

[10] Rehman J (2004). Secretion of angiogenic and antiapoptotic factors by human adipose stromal cells. Circulation.

[11] Linero I, Chaparro O (2014). Paracrine effect of mesenchymal stem cells derived from human adipose tissue in bone regeneration. PLoS ONE.

[12] Wang L (2017). Exosomes secreted by human adipose mesenchymal stem cells promote scarless cutaneous repair by regulating extracellular matrix remodelling. Sci. Rep..

[13] Dalloul A (2013). Hypoxia and visualization of the stem cell niche. Methods Mol. Biol..

[14] Chung HM, Won CH, Sung JH (2009). Responses of adipose-derived stem cells during hypoxia: enhanced skin-regenerative potential. Expert Opin. Biol. Ther..

[15] Lee EY (2009). Hypoxia-enhanced wound-healing function of adipose-derived stem cells: increase in stem cell proliferation and up-regulation of VEGF and bFGF. Wound Repair Regen..

[16] Stubbs SL (2012). Hypoxic preconditioning enhances survival of human adipose-derived stem cells and conditions endothelial cells in vitro. Stem Cells Dev..

[17] Wang F (2018). Hypoxia enhances differentiation of adipose tissue-derived stem cells toward the smooth muscle phenotype. Int. J. Mol. Sci..

[18] Han Y, Ren J, Bai Y, Pei X (2019). Exosomes from hypoxia-treated human adipose-derived mesenchymal stem cells enhance angiogenesis through VEGF/VEGF-R. Int. J. Biochem. Cell Biol..

[19] Frazier TP, Gimble JM, Kheterpal I, Rowan BG (2013). Impact of low oxygen on the secretome of human adipose-derived stromal/stem cell primary cultures. Biochimie.

[20] Wang X (2015). Hypoxia precondition promotes adipose-derived mesenchymal stem cells based repair of diabetic erectile dysfunction via augmenting angiogenesis and neuroprotection. PLoS ONE.

[21] Riis S (2016). Mass spectrometry analysis of adipose-derived stem cells reveals a significant effect of hypoxia on pathways regulating extracellular matrix. Stem Cell Res. Ther..

[22] Schwarz C (2012). Effects of different media on proliferation and differentiation capacity of canine, equine and porcine adipose derived stem cells. Res. Vet. Sci..

[23] Feyen DA (2016). Isolation of pig bone marrow-derived mesenchymal stem cells. Methods Mol Biol.

[24] Bukowska J, Ziecik AJ, Laguna J, Gawronska-Kozak B, Bodek G (2015). The importance of the canonical wnt signaling pathway in the porcine endometrial stromal stem/progenitor cells: implications for regeneration. Stem Cells Dev..

[25] Bui HT (2014). Identification and characterization of putative stem cells in the adult pig ovary. Development.

[26] Eirin A (2017). Integrated transcriptomic and proteomic analysis of the molecular cargo of extracellular vesicles derived from porcine adipose tissue-derived mesenchymal stem cells. PLoS ONE.

[27] James I (2018). Adipose stem cells enhance excisional wound healing in a porcine model. J. Surg. Res..

[28] Bourin P (2013). Stromal cells from the adipose tissue-derived stromal vascular fraction and culture expanded adipose tissue-derived stromal/stem cells: a joint statement of the International Federation for Adipose Therapeutics and Science (IFATS) and the International Society for Cellular Therapy (ISCT). Cytotherapy.

[29] Zhang S (2016). Identification and characterization of pig adipose-derived progenitor cells. Can. J. Vet. Res..

[30] Niada S (2013). Porcine adipose-derived stem cells from buccal fat pad and subcutaneous adipose tissue for future preclinical studies in oral surgery. Stem Cell Res. Ther..

[31] Bukowska J (2020). Safety and efficacy of human adipose-derived stromal/stem cell therapy in an immunocompetent murine pressure ulcer model. Stem Cells Dev..

[32] Casado JG (2012). Comparative phenotypic and molecular characterization of porcine mesenchymal stem cells from different sources for translational studies in a large animal model. Vet. Immunol. Immunopathol..

[33] Bruckner S (2014). A fat option for the pig: hepatocytic differentiated mesenchymal stem cells for translational research. Exp. Cell Res..

[34] Lee AY (2015). Comparative studies on proliferation, molecular markers and differentiation potential of mesenchymal stem cells from various tissues (adipose, bone marrow, ear skin, abdominal skin, and lung) and maintenance of multipotency during serial passages in miniature pig. Res. Vet. Sci..

[35] Ock SA (2016). Comparison of immunomodulation properties of porcine mesenchymal stromal/stem cells derived from the bone marrow, adipose tissue, and dermal skin tissue. Stem Cells Int..

[36] Hong WX (2014). The role of hypoxia-inducible factor in wound healing. Adv. Wound Care.

[37] Sipila KH (2018). Proline hydroxylation in collagen supports integrin binding by two distinct mechanisms. J. Biol. Chem..

[38] Gauglitz GG, Korting HC, Pavicic T, Ruzicka T, Jeschke MG (2011). Hypertrophic scarring and keloids: pathomechanisms and current and emerging treatment strategies. Mol. Med..

[39] Li Q (2019). Proteomic analysis of human periodontal ligament cells under hypoxia. Proteome Sci..

[40] Zhang K (2017). Proteome analysis of hypoxic glioblastoma cells reveals sequential metabolic adaptation of one-carbon metabolic pathways. MCP.

[41] Arthur SA, Blaydes JP, Houghton FD (2019). Glycolysis regulates human embryonic stem cell self-renewal under hypoxia through HIF-2alpha and the glycolytic sensors CTBPs. Stem cell reports.

[42] Liu Y, Yuan X, Munoz N, Logan TM, Ma T (2019). Commitment to aerobic glycolysis sustains immunosuppression of human mesenchymal stem cells. Stem Cells Transl. Med..

[43] Garrido C (2006). Heat shock proteins 27 and 70: anti-apoptotic proteins with tumorigenic properties. Cell Cycle.

[44] Brodarac A (2015). Susceptibility of murine induced pluripotent stem cell-derived cardiomyocytes to hypoxia and nutrient deprivation. Stem Cell Res. Ther..

[45] Oberringer M (1995). Differential expression of heat shock protein 70 in well healing and chronic human wound tissue. Biochem. Biophys. Res. Commun..

[46] Kovalchin JT (2006). In vivo delivery of heat shock protein 70 accelerates wound healing by up-regulating macrophage-mediated phagocytosis. Wound Repair Regener..

[47] Bradley E, Bieberich E, Mivechi NF, Tangpisuthipongsa D, Wang G (2012). Regulation of embryonic stem cell pluripotency by heat shock protein 90. Stem Cells.

[48] Li W (2007). Extracellular heat shock protein-90alpha: linking hypoxia to skin cell motility and wound healing. EMBO J..

[49] Lee WJ (2015). Heat shock protein 90 inhibitor decreases collagen synthesis of keloid fibroblasts and attenuates the extracellular matrix on the keloid spheroid model. Plast. Reconstr. Surg..

[50] Vogler M (2013). Hypoxia modulates fibroblastic architecture, adhesion and migration: a role for HIF-1alpha in cofilin regulation and cytoplasmic actin distribution. PLoS ONE.

[51] Waschke J, Curry FE, Adamson RH, Drenckhahn D (2005). Regulation of actin dynamics is critical for endothelial barrier functions. Am. J. Physiol. Heart Circ. Physiol..

[52] Wu C (2007). Focal adhesion: a focal point in current cell biology and molecular medicine. Cell Adhes. Migr..

[53] Kim YM (2008). Angiotensin II-induced differentiation of adipose tissue-derived mesenchymal stem cells to smooth muscle-like cells. Int. J. Biochem. Cell Biol..

[54] Rodriguez LV (2006). Clonogenic multipotent stem cells in human adipose tissue differentiate into functional smooth muscle cells. Proc. Natl. Acad. Sci. U.S.A..

[55] Ricco S (2013). Allogeneic adipose tissue-derived mesenchymal stem cells in combination with platelet rich plasma are safe and effective in the therapy of superficial digital flexor tendonitis in the horse. Int. J. Immunopathol. Pharmacol..

[56] Enciso N, Avedillo L, Fermin ML, Fragio C, Tejero C (2020). Regenerative potential of allogeneic adipose tissue-derived mesenchymal cells in canine cutaneous wounds. Acta Vet. Scand..

[57] Ekser B (2012). Clinical xenotransplantation: the next medical revolution?. Lancet.

[58] Yu G (2011). Adipogenic differentiation of adipose-derived stem cells. Methods Mol. Biol..

[59] Staszkiewicz J (2010). Cell growth characteristics, differentiation frequency, and immunophenotype of adult ear mesenchymal stem cells. Stem Cells Dev..

[60] Bradford MM (1976). A rapid and sensitive method for the quantitation of microgram quantities of protein utilizing the principle of protein-dye binding. Anal. Biochem..

[61] Kanehisa M (2019). Toward understanding the origin and evolution of cellular organisms. Protein Sci..

[62] Kanehisa M, Goto S (2000). KEGG: kyoto encyclopedia of genes and genomes. Nucleic Acids Res..

[63] Kanehisa M, Sato Y, Furumichi M, Morishima K, Tanabe M (2019). New approach for understanding genome variations in KEGG. Nucleic Acids Res..

[64] Szklarczyk D (2019). STRING v11: protein-protein association networks with increased coverage, supporting functional discovery in genome-wide experimental datasets. Nucleic Acids Res..

[65] Bukowska J (2018). Effect of TGFbeta1, TGFbeta3 and keratinocyte conditioned media on functional characteristics of dermal fibroblasts derived from reparative (Balb/c) and regenerative (Foxn1 deficient; nude) mouse models. Cell Tissue Res..

[66] Gawronska-Kozak B, Kirk-Ballard H (2013). Cyclosporin A reduces matrix metalloproteinases and collagen expression in dermal fibroblasts from regenerative FOXN1 deficient (nude) mice. Fibrogenesis Tissue Repair.

