# Supplementary material for: The effect of hypoxia on the proteomic signature of pig adipose-derived stromal/stem cells (pASCs)
Source: Sci Rep. 2020 Nov 18;10:20035. doi: 10.1038/s41598-020-76796-7 (PMC7676232; doi:10.1038/s41598-020-76796-7)
Supplement: Supplementary file 1 — Supplementary information. [file 41598_2020_76796_MOESM1_ESM.docx]

**The effect of hypoxia on the proteomic signature of pig adipose-derived stromal/stem cells (pASCs)**

Joanna Bukowska, Mariola Słowińska, Patrycja Cierniak, Marta Kopcewicz, Katarzyna Walendzik, Trivia Frazier, Barbara Gawrońska-Kozak

# **Supplementary Information**

# **Supplementary Results**

## **Supplementary Table. S1.**

**Supplementary Figure S1.**

## **Supplementary Table. S2.**

## **Supplementary Table. S3.**

## **Supplementary Table. S4.**

## **Supplementary Table. S5a.**

## **Supplementary Table. S5b.**

## **Supplementary Table. S6a.**

## **Supplementary Table. S6b**

## **Supplementary Table. S7.**

## **Supplementary Table. S8.**

## **Supplementary Table. S9a.**

## **Supplementary Table. S9b.**

## **Supplementary Figure S2.**

## **Supplementary Figure Legend S2.**

# **Supplementary Information Methods**

## **Proteomics**

**Supplementary Table S10.**

## **RNA isolation and real-time PCR**

**Supplementary Table S11.**

## **Collagen gel contraction assay**

## ***In vitro* wound migration assay**

# **Supplementary References**

## **Supplementary Table. S1.**

Presentation of statistical analysis for pASCs growth kinetics (statistic refers to Fig.1B in the main body of manuscript).

| Table S4. | **Estimate** | **Std. Error** | **t value** | **Pr(>\|t\|)** |
| --- | --- | --- | --- | --- |
| **(Intercept)** | 3.052 | 0.805 | 3.790 | 0.001 |
| **gestosc2.5x105** | 0.796 | 1.139 | 0.699 | **0.493** |
| **gestosc5.0x104** | -0.909 | 1.074 | -0.846 | **0.407** |

## **Supplementary Table. S2.**

List of pASCs proteins differentially expressed in response to hypoxia (1% O_2_) or normoxia (21% O_2_) (n = 4 in each group; *p* < 0.05). Up - arrows denote a higher abundance of proteins in hypoxia – treated pASCs, while down - arrows indicate proteins with increased abundance in pASCs cultured under normoxia. Asterisk (*) indicates proteins that show the expression fold-change ≤ 1.10.

| **Pos.** | **Protein name (*Organism*)** | **Gene ID** | **Accession**  **number** | **Calculated**  **MW/pI** | **Protein score** | **Sequence coverage**  **%** | **No of peptides** | **Paired T-test** | **Paired Av Ratio** |
| --- | --- | --- | --- | --- | --- | --- | --- | --- | --- |
| **Up-regulated upon hypoxia (1%O_2_)** | | | | | | | | | |
| 1408 ↑ | protein NDRG1 *[Sus scrofa]* | **NDRG1** | XP_020944534 | 42187/5.42 | 189 | 29 | 3 | 0.018 | **-1.89** |
| 2473 ↑ | translocon-associated protein subunit delta isoform X2  *[Sus scrofa]* | **SSR4** | XP_005674066 | 19017/5.49 | 269 | 32 | 3 | 0.015 | **-1.78** |
| 938 ↑ | prolyl 4-hydroxylase subunit alpha-1 precursor *[Sus scrofa]* | **P4HA1** | NP_001090904 | 61239/5.70 | 696 | 39 | 6 | 0.014 | **-1.25** |
| 1222 ↑ | 4-trimethylaminobutyraldehyde dehydrogenase isoform X1  *[Sus scrofa]* | **ALDH9A1** | XP_005663205 | 65826/8.65 | 306 | 22 | 4 | 0.0028 | **-1.25** |
| 1590 ↑ | fructose-bisphosphate aldolase C *[Sus scrofa]* | **ALDOC** | NP_001230857 | 39751/6.21 | 208 | 38 | 2 | 0.0068 | **-1.23** |
| 950 ↑ | ERO1-like protein alpha precursor *[Sus scrofa]* | **ERO1A** | NP_001131099 | 55101/5.81 | 233 | 39 | 2 | 0.0036 | **-1.21** |
| 1417 ↑ | gamma-enolase isoform X1  *[Sus scrofa]* | **ENO2** | XP_005652652 | 47509/4.90 | 191 | 25 | 2 | 0.0037 | **-1.20** |
| 485 ↑ | prelamin-A/C *[Sus scrofa]* | **LMNA** | NP_001104727 | 74416/6.73 | 495 | 48 | 5 | 0.0078 | **-1.19** |
| 516 ↑ | zyxin *[Sus scrofa]* | **ZYX** | XP_003134640 | 62353/6.37 | 466 | 32 | 4 | 0.0087 | **-1.19** |
| 566 ↑ | vimentin isoform X1 *[Sus scrofa]* | **VIM** | XP_005668163 | 53692/5.06 | 1170 | 65 | 10 | 0.025 | **-1.18** |
| 2121 ↑ | PREDICTED: phosphoglycerate mutase 1-like *[Sus scrofa]* | **PGAM1** | XP_003483583 | 28900/6.67 | 674 | 70 | 5 | 0.023 | **-1.18** |
| 119 ↑ | L-lactate dehydrogenase A chain *[Sus scrofa]* | **LDHA** | NP_001165834 | 36852/8.18 | 189 | 25 | 2 | 0.035 | **-1.17** |
| 339 ↑ | vimentin isoform X1 *[Sus scrofa]* | **VIM** | XP_005668163 | 53692/5.06 | 842 | 63 | 9 | 0.033 | **-1.17** |
| 2069 ↑ | triosephosphate isomerase 1  *[Sus scrofa]* | **TPI1** | BAI48105 | 26879/6.54 | 675 | 54 | 6 | 0.0061 | **-1.16** |
| 560 ↑ | vimentin isoform X1 *[Sus scrofa]* | **VIM** | XP_005668163 | 53692/5.06 | 1050 | 63 | 9 | 0.015 | **-1.15** |
| 2195 ↑ | peroxiredoxin-6 *[Sus scrofa]* | **PRDX6** | NP_999573 | 25078/5.73 | 693 | 71 | 7 | 0.0088 | **-1.15** |
| 504 ↑ | prelamin-A/C *[Sus scrofa]* | **LMNA** | NP_001104727 | 74416/6.73 | 207 | 42 | 2 | 0.00019 | **-1.14** |
| 50 ↑ | L-lactate dehydrogenase A chain *[Sus scrofa]* | **LDHA** | NP_001165834 | 36852/8.18 | 307 | 34 | 2 | 0.049 | **-1.13** |
| 367 ↑ | vinculin isoform X3 *[Sus scrofa]* | **VCL** | XP_005671131 | 117246/5.83 | 127 | 9 | 2 | 0.044 | **-1.13** |
| 400 ↑ | vimentin isoform X1 *[Sus scrofa]* | **VIM** | XP_005668163 | 53692/5.06 | 958 | 66 | 9 | 0.020 | **-1.13** |
| 667 ↑ | sorting nexin-18 isoform X1 *[Sus scrofa]* | **SNX18** | XP_020932615 | 69295/5.97 | 205 | 25 | 3 | 0.0071 | **-1.13** |
| 868 ↑ | heat shock 70 kDa protein 1B *[Sus scrofa]* | **HSP70.2** | NP_998931 | 70340/5.60 | 878 | 57 | 8 | 0.0039 | **-1.13** |
| 1641 ↑ | fructose-bisphosphate aldolase A isoform X4 *[Sus scrofa]* | **ALDOA** | XP_020943659 | 39852/8.49 | 431 | 48 | 5 | 0.00094 | **-1.13** |
| 1822 ↑ | glyceraldehyde-3-phosphate dehydrogenase *[Sus scrofa]* | **GAPDH** | NP_001193288 | 36041/8.51 | 501 | 42 | 4 | 0.027 | **-1.13** |
| 1823 ↑ | glyceraldehyde-3-phosphate dehydrogenase (phosphorylating) (EC 1.2.1.12) *[Sus scrofa]* | **GAPDH** | pir\|\|DEPGG3 | 35914/6.90 | 255 | 32 | 3 | 0.0035 | **-1.13** |
| 1827 ↑ | glyceraldehyde-3-phosphate dehydrogenase *[Sus scrofa]* | **GAPDH** | NP_001193288 | 36041/8.51 | 434 | 36 | 4 | 0.0067 | **-1.13** |
| 2081↑ | triosephosphate isomerase 1  *[Sus scrofa]* | **TPI1** | BAI48105 | 26879/6.54 | 1010 | 81 | 8 | 0.012 | **-1.13** |
| 2409 ↑ | transgelin-2 *[Sus scrofa]* | **TAGLN2** | XP_003125720 | 22534/8.41 | 601 | 63 | 6 | 0.0060 | **-1.13** |
| 2645 ↑ | peptidyl-prolyl cis-trans isomerase FKBP1A *[Sus scrofa]* | **FKBP1A** | NP_001033089 | 11945/7.88 | 222 | 41 | 3 | 0.0038 | **-1.13** |
| 736 ↑ | src substrate cortactin isoform X1 *[Sus scrofa]* | **CTTN** | XP_020938306 | 61131/5.23 | 116 | 21 | 2 | 0.038 | **-1.12** |
| 983 ↑ | PREDICTED: UDP-N-acetylhexosamine pyrophosphorylase *[Sus scrofa]* | **UAP1** | XP_001928678 | 57426/5.85 | 218 | 17 | 2 | 0.021 | **-1.12** |
| 1211 ↑ | UTP--glucose-1-phosphate uridylyltransferase *[Sus scrofa]* | **UGP2** | NP_999145 | 57089/7.69 | 474 | 32 | 5 | 0.012 | **-1.12** |
| 1413 ↑ | phosphoglycerate kinase 1  *[Sus scrofa]* | **PGK1** | NP_001093402 | 44929/8.02 | 704 | 60 | 7 | 0.010 | **-1.12** |
| 1645 ↑ | fructose-bisphosphate aldolase A isoform X4 *[Sus scrofa]* | **ALDOA** | XP_020943659 | 39852/8.49 | 282 | 48 | 2 | 0.0040 | **-1.12** |
| 1828 ↑ | glyceraldehyde-3-phosphate dehydrogenase (phosphorylating) (EC 1.2.1.12) *[Sus scrofa]* | **GAPDH** | DEPGG3 | 35914/6.90 | 470 | 42 | 5 | 0.0046 | **-1.12** |
| 1939 ↑ | L-lactate dehydrogenase A chain *[Sus scrofa]* | **LDHA** | NP_001165834 | 36852/8.18 | 469 | 34 | 5 | 0.043 | **-1.12** |
| 1613 ↑ | mitogen-activated protein kinase 1 *[Sus scrofa]* | **MAPK1** | NP_001185851 | 41677/6.50 | 256 | 24 | 3 | 0.036 | **-1.11** |
| 1646 ↑ | fructose-bisphosphate aldolase A isoform X4 *[Sus scrofa]* | **ALDOA** | XP_020943659 | 39852/8.49 | 525 | 50 | 6 | 0.0015 | **-1.11** |
| 1819 ↑ | glyceraldehyde-3-phosphate dehydrogenase (phosphorylating) (EC 1.2.1.12) *[Sus scrofa]* | **GAPDH** | DEPGG3 | 35914/6.90 | 420 | 37 | 3 | 0.037 | **-1.11** |
| 2161 ↑ | triosephosphate isomerase 1  *[Sus scrofa]* | **TPI1** | BAI48105 | 26879/6.54 | 537 | 70 | 5 | 0.030 | **-1.11** |
| 2411 ↑ | transgelin-2 *[Sus scrofa]* | **TAGLN2** | XP_003125720 | 22534/8.41 | 637 | 73 | 7 | 0.024 | **-1.11** |
| 3047 ↑ | alpha-enolase isoform X2  *[Sus scrofa]* | **ENO1** | XP_020950939 | 47801/6.17 | 481 | 42 | 3 | 0.0022 | **-1.11** |
| 3051 ↑ | caldesmon isoform X10  *[Sus scrofa]* | **CALD1** | XP_020934831 | 62291/6.04 | 434 | 30 | 4 | 0.043 | **-1.11** |
| 3052 ↑ | alpha-enolase isoform X1  *[Sus scrofa]* | **ENO1** | XP_020950937 | 47600/6.44 | 953 | 51 | 8 | 0.018 | **-1.11** |
| *939 ↑ | LOW QUALITY PROTEIN: plastin-3 *[Sus scrofa]* | **PLS3** | XP_001925971 | 71220/5.35 | 419 | 36 | 4 | 0.048 | **-1.10** |
| *1197 ↑ | tubulin alpha-1B chain *[Sus scrofa]* | **TUBA1B** | NP_001038009 | 50804/4.94 | 912 | 61 | 7 | 0.048 | **-1.09** |
| *2020 ↑ | chloride intracellular channel protein 1 *[Sus scrofa]* | **CLIC1** | XP_020954120 | 27303/5.17 | 982 | 68 | 9 | 0.024 | **-1.09** |
| *1356↑ | alpha-enolase isoform X1  *[Sus scrofa]* | **ENO1** | XP_020950937 | 47600/6.44 | 799 | 51 | 6 | 0.015 | **-1.08** |
| *1912 ↑ | tropomyosin alpha-4 chain  *[Sus scrofa]* | **TPM4** | NP_999500 | 28619/4.76 | 815 | 56 | 8 | 0.022 | **-1.08** |
| *1974 ↑ | annexin A5 *[Sus scrofa]* | **ANXA5** | XP_003129266 | 36169/4.94 | 694 | 68 | 7 | 0.0046 | **-1.08** |
| *1021 ↑ | pyruvate kinase PKM isoform X8 *[Sus scrofa]* | **PKM** | XP_001929104 | 58411/7.96 | 562 | 39 | 5 | 0.049 | **-1.07** |
| *1031 ↑ | pyruvate kinase PKM isoform X8 *[Sus scrofa]* | **PKM** | XP_001929104 | 58411/7.96 | 873 | 63 | 7 | 0.0096 | **-1.07** |
| *1081 ↑ | heterogeneous nuclear ribonucleoprotein K *[Sus scrofa]* | **HNRNPK** | NP_001254774 | 51221/5.33 | 410 | 22 | 4 | 0.029 | **-1.07** |
| *1243↑ | polymerase I and transcript release factor *[Sus scrofa]* | **CAVIN1** | XP_012299524 | 43556/5.50 | 213 | 19 | 3 | 0.042 | **-1.07** |
| *1555 ↑ | keratin, type I cytoskeletal 19  *[Sus scrofa]* | **KRT19** | XP_003131485 | 44186/5.05 | 388 | 42 | 2 | 0.042 | **-1.07** |
| *1855 ↑ | annexin A2 isoform X1 *[Sus scrofa]* | **ANXA2** | XP_005659594 | 38840/6.92 | 1050 | 71 | 9 | 0.044 | **-1.07** |
| *483 ↑ | alpha-actinin-1 *[Sus scrofa]* | **Actn1** | NP_001229990 | 103111/5.33 | 487 | 41 | 6 | 0.042 | **-1.06** |
| *1162 ↑ | PREDICTED: tubulin alpha-1A chain isoform 2 *[Sus scrofa]* | **TUBA1A** | XP_003481619 | 46781/4.96 | 766 | 62 | 7 | 0.035 | **-1.06** |
| *1971 ↑ | annexin A5 *[Sus scrofa]* | **ANXA5** | XP_003129266 | 36169/4.94 | 409 | 54 | 4 | 0.0058 | **-1.06** |
| *961 ↑ | PREDICTED: pyruvate kinase isozymes M1/M2 isoform 1 *[Sus scrofa]* | **PKM** | XP_001929104 | 58411/7.96 | 309 | 37 | 3 | 0.0054 | **-1.05** |
| *3048 ↑ | PREDICTED: 14-3-3 protein epsilon-like *[Sus scrofa]* | **YWHAE** | XP_003361720 | 26780/5.31 | 309 | 43 | 3 | 0.0050 | **-1.05** |
| *2573 ↑ | peptidyl-prolyl cis-trans isomerase A *[Sus scrofa]* | **PPIA** | NP_999518 | 18086/8.34 | 342 | 44 | 3 | 0.018 | **-1.03** |
| **Down-regulated upon hypoxia (1%O_2_)** | | | | | | | | | |
| 3046 ↓ | collagen alpha-2(I) chain precursor *[Sus scrofa]* | **COL1A2** | NP_001230584 | 129652/9.13 | 259 | 32 | 3 | 0.037 | **1.38** |
| 315 ↓ | elongation factor 2 isoform X1 *[Sus scrofa]* | **EEF2** | XP_003354050 | 96262/6.41 | 437 | 30 | 3 | 0.047 | **1.31** |
| 2287 ↓ | eukaryotic translation initiation factor 4H isoform 2 *[Sus scrofa]* | **EIF4H** | NP_001230377 | 25224/7.82 | 321 | 35 | 2 | 0.0038 | **1.29** |
| 811 ↓ | stress-70 protein, mitochondrial *[Sus scrofa]* | **HSPA9** | XP_005661752 | 73890/5.81 | 1060 | 43 | 10 | 0.012 | **1.27** |
| 836 ↓ | probable ATP-dependent RNA helicase DDX5 isoform X1 *[Sus scrofa]* | **DDX5** | XP_020922417 | 74007/9.25 | 211 | 26 | 3 | 0.014 | **1.26** |
| 433 ↓ | elongation factor 2 isoform X2 *[Sus scrofa]* | **EEF2** | XP_020939748 | 94875/6.41 | 685 | 49 | 8 | 0.025 | **1.21** |
| 1148 ↓ | aldehyde dehydrogenase, mitochondrial precursor *[Sus scrofa]* | **ALDH2** | NP_001038076 | 57341/6.43 | 525 | 28 | 4 | 0.015 | **1.19** |
| 1004 ↓ | catalase *[Sus scrofa]* | **CAT** | NP_999466 | 60177/6.60 | 406 | 40 | 5 | 0.016 | **1.18** |
| 676 ↓ | heat shock 90kD protein 1, beta *[Sus scrofa]* | **HSP90AB1** | NP_001231362 | 83543/4.96 | 1090 | 43 | 10 | 0.040 | **1.16** |
| 1190 ↓ | ATP synthase subunit alpha, mitochondrial *[Sus scrofa]* | **ATP5F1A** | NP_001172071 | 59765/9.21 | 643 | 46 | 5 | 0.0022 | **1.16** |
| 1057 ↓ | 60 kDa heat shock protein, mitochondrial *[Sus scrofa]* | **HSPD1** | NP_001241645 | 61058/5.70 | 702 | 47 | 7 | 0.026 | **1.14** |
| 1909 ↓ | pyrroline-5-carboxylate reductase 1, mitochondrial isoform X3  *[Sus scrofa]* | **PYCR1** | XP_020922074 | 33512/6.55 | 220 | 42 | 2 | 0.0018 | **1.14** |
| 749 ↓ | lysine--tRNA ligase isoform X1 *[Sus scrofa]* | **KARS** | XP_003355791 | 71842/6.40 | 174 | 18 | 3 | 0.023 | **1.13** |
| 328 ↓ | elongation factor 2 isoform X1 *[Sus scrofa]* | **EEF2** | XP_003354050 | 96262/6.41 | 556 | 28 | 6 | 0.0079 | **1.12** |
| 1054 ↓ | dihydrolipoyl dehydrogenase, mitochondrial precursor *[Sus scrofa]* | **DLD** | NP_999227 | 54721/7.59 | 146 | 22 | 2 | 0.023 | **1.12** |
| 839 ↓ | phosphoenolpyruvate carboxykinase [GTP], mitochondrial *[Sus scrofa]* | **PCK2** | NP_001155225 | 71387/8.34 | 346 | 29 | 3 | 0.0028 | **1.11** |
| 1161 ↓ | glutamate dehydrogenase 1, mitochondrial *[Sus scrofa]* | **GLUD1** | NP_001231430 | 61668/8.03 | 285 | 32 | 3 | 0.020 | **1.11** |
| 2103 ↓ | enoyl-CoA hydratase, mitochondrial *[Sus scrofa]* | **ECHS1** | NP_001177104 | 31558/8.81 | 184 | 29 | 2 | 0.0075 | **1.11** |
| *674 ↓ | PREDICTED: ATP-dependent RNA helicase DDX3X, partial *[Sus scrofa]* | **Ddx3x** | XP_008507782 | 71993/7.31 | 512 | 38 | 3 | 0.0085 | **1.10** |
| *1151 ↓ | glutamate dehydrogenase 1, mitochondrial *[Sus scrofa]* | **GLUD1** | NP_001231430 | 61668/8.03 | 288 | 27 | 2 | 0.026 | **1.10** |
| *1479 ↓ | elongation factor 1-alpha 1  *[Sus scrofa]* | **EEF1A1** | NP_001090887 | 50451/9.10 | 241 | 20 | 3 | 0.031 | **1.10** |
| *327 ↓ | staphylococcal nuclease domain-containing protein 1 *[Sus scrofa]* | **SND1** | XP_020934292 | 102520/6.72 | 799 | 43 | 6 | 0.014 | **1.09** |
| *684 ↓ | PREDICTED: polyadenylate-binding protein 1 *[Sus scrofa]* | **Pabpc1** | XP_001927782 | 70854/9.52 | 201 | 19 | 3 | 0.037 | **1.09** |
| *1473 ↓ | serpin H1 precursor *[Sus scrofa]* | **SERPINH1** | NP_001231061 | 46648/8.91 | 371 | 29 | 4 | 0.034 | **1.09** |
| *543 ↓ | glutamine--tRNA ligase *[Sus scrofa]* | **QARS** | XP_020926311 | 88421/6.49 | 361 | 33 | 4 | 0.019 | **1.06** |
| *431 ↓ | serpin H1 precursor *[Sus scrofa]* | **SERPINH1** | NP_001231061 | 46648/8.91 | 401 | 39 | 4 | 0.043 | **1.05** |
| *432 ↓ | elongation factor 2 *[Sus scrofa]* | **EEF2** | XP_003354050 | 96262/6.41 | 365 | 22 | 3 | 0.049 | **1.05** |
| *1440 ↓ | proliferation-associated protein 2G4 isoform X2 *[Sus scrofa]* | **PA2G4** | XP_020947459 | 38306/7.15 | 507 | 50 | 5 | 0.041 | **1.05** |
| *1441 ↓ | put. 26S protease subunit  *[Sus scrofa]* | **PSMC5**  **(TBP10)** | CAA61864 | 44927/8.23 | 540 | 44 | 6 | 0.012 | **1.05** |
| *1863 ↓ | voltage-dependent anion-selective channel protein 2 isoform X1  *[Sus scrofa]* | **VDAC2** | XP_020927834 | 32098/7.48 | 521 | 62 | 5 | 0.044 | **1.04** |
| *308 ↓ | cytoskeletal beta actin, partial *[Sus scrofa]* | **ACTB** | AAS55927 | 45162/5.55 | 118 | 23 | 2 | 0.050 | **1.03** |

**
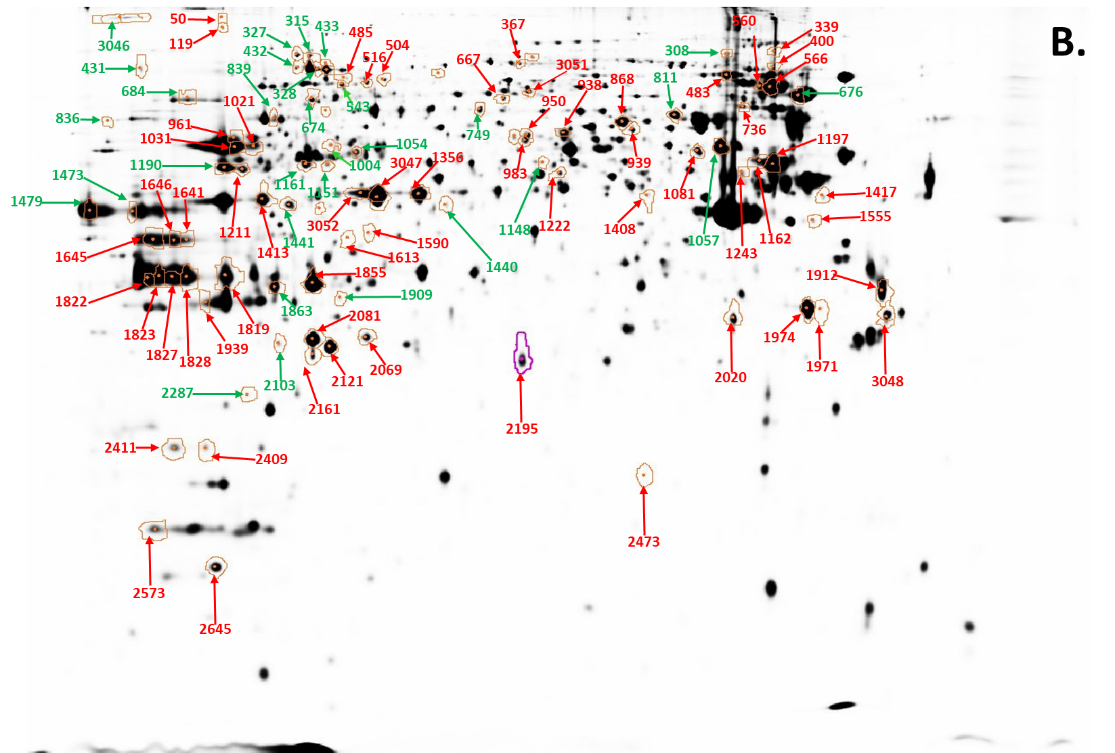
Supplementary Figure S1.**

**
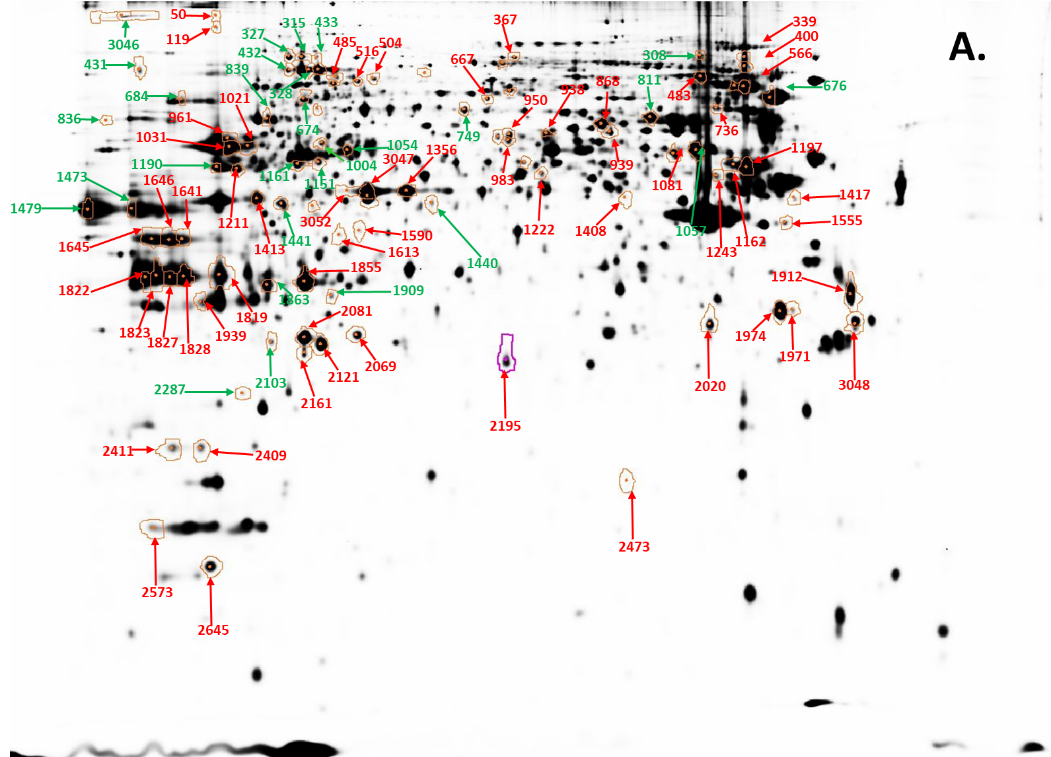
**

**
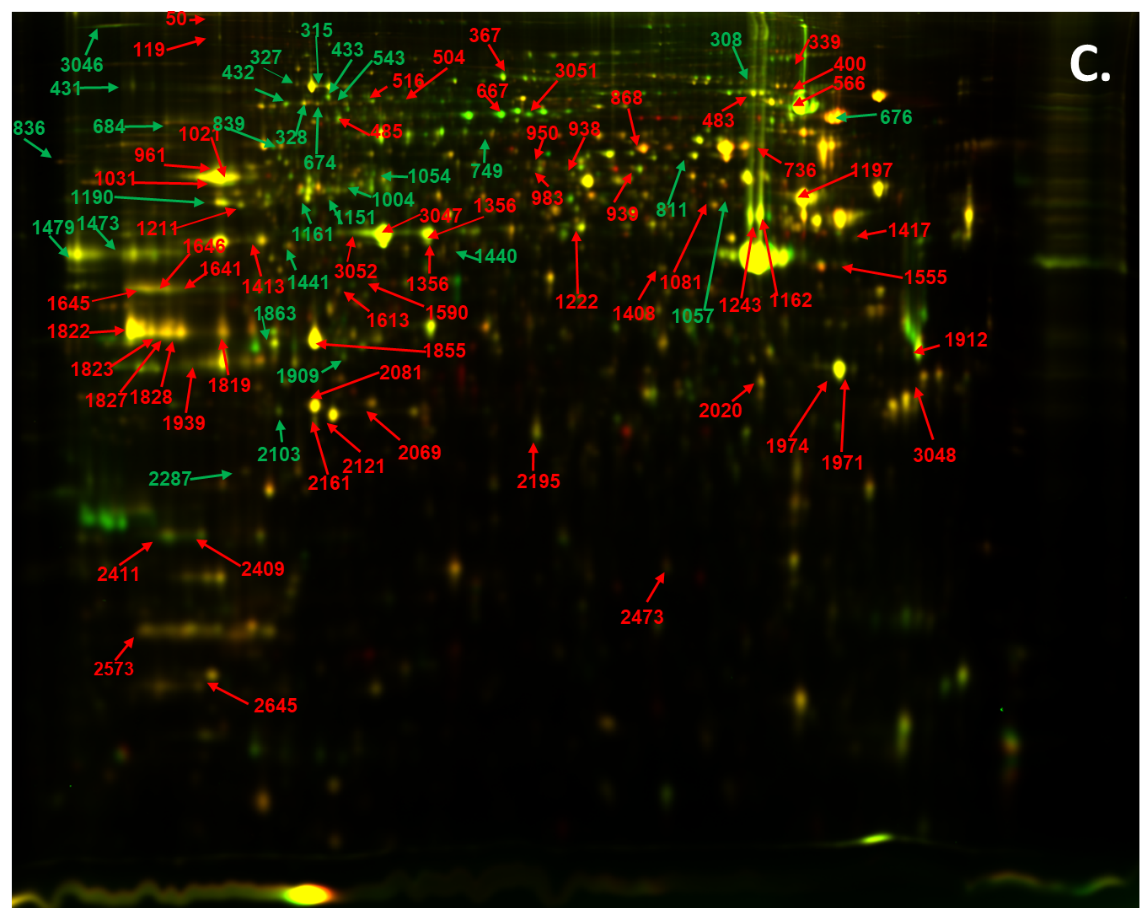
Supplementary Figure S1.**

Two-dimensional difference gel electrophoresis (2D-DIGE) analysis of pASCs cultured 24 h in different oxygen environments. (A) Single-channel image of proteins from pASCs cultured in normoxia (21% O_2_). B) Single-channel image of proteins from pASCs exposed to hypoxia (1% O_2_). C) Overlay of Cy3 and Cy5 channel images according to the arrangement presented in Supplementary Table 10 for gel no 4. Numbered protein spots (red) indicate the proteins identified from 2D-DIGE that are more abundant in pASCs upon normoxia (21% O_2_) and (green) correspond to the identified spots that are more abundant in pASCs exposed to hypoxia (1% O_2_). Results of spot identification are presented in Supplementary Table S2. Panels A, B originated directly from Biological Variation Analysis workspace of DeCyder Differential Analysis Software. Panel C came from Image Quant TL. The differentiated proteins were marked manually.

## **Supplementary Table. S3.**

List of pASCs proteins up-regulated upon hypoxia (1% O_2_). Gene ontology (GO) annotation was used to determine the **“**Biological processes”, “Molecular function”, “Cellular component”, and “Protein class” of the proteins; (n = 4; *p* < 0.05; proteins demonstrated fold change >1.10 are included).

| **Proteins up-regulated upon hypoxia** | | |
| --- | --- | --- |
| **GO classification** | **%** | **Protein symbol** |
| **Biological Processes** | | |
| *cellular process* | 36.4 | PGK1, TPI1, FKBP1A, UAP1, CTTN, NDRG1, ZYX, ERO1A, MAPK1, ALDOC, PRDX6, ALDOA |
| *metabolic process* | 21.2 | TPI1, UAP1, MAPK1, PGK1, FKBP1A, ALDOC, ALDOA |
| *biological regulation* | 18.2 | PGK1, MAPK1, ZYX, CTTN, NDRG1, PRDX6 |
| *localization* | 3.0 | PGK1 |
| *response to stimulus* | 9.1 | MAPK1,ZYX, NDRG1 |
| *cellular component organization or biogenesis* | 3.0 | CTTN |
| *signaling* | 3.0 | ZYX, MAPK1, NDRG1 |
| **Molecular function** | | |
| *catalytic activity* | 81.8 | PGK1, FKBP1A, UAP1, ALDH9A1, MAPK1, TPI1 |
| *binding* | 18.2 | PGK1, CTTN |
| **Cellular component** | | |
| *cell* | 50.0 | PGK1, TPI1, FKBP1A, PRDX6, MAPK1, NDRG1, ZYX, ERO1A, CTTN, ALDOC, ALDOA |
| *organelle* | 10.5 | CTTN, MAPK1 ERO1A, ZYX |
| *supramolecular complex* | 9.1 | ZYX, CTTN |
| *cell junction* | 9.1 | ZYX, CTTN |
| *membrane* | 9.1 | CTTN, ERO1A |
| *protein-containing complex* | 4.5 | CTTN |
| **Protein class** | | |
| *metabolite interconversion enzyme* | 56.3 | GAPDH, LDHA, ENO2, ERO1A, ENO1, PRDX6, ALDH9A1, PGK1, TPI1 |
| *cytoskeletal protein* | 25.0 | TAGLN2, ZYX, CALD1, CTTN |
| *protein modifying enzyme* | 12.5 | MAPK1, NDRG1 |
| *chaperone* | 3.6 | FKBP1A |

## **Supplementary Table. S4.**

List of pASCs proteins down-regulated upon hypoxia (1% O_2_). Gene ontology (GO) annotation was used to determine the **“**Biological processes”, “Molecular function”, “Cellular component”, and “Protein class” of the proteins; (n = 4; *p* < 0.05; proteins demonstrated fold change >1.10 are included).

| **Proteins down-regulated upon hypoxia** | | |
| --- | --- | --- |
| **GO classification** | **%** | **Protein symbol** |
| **Biological Processes** | | |
| *cellular process* | 38.1 | CAT, HSPD1, HSP90AB1, PCK2, EEF2, PYCR1, KARS,  ECHS1 |
| *metabolic process* | 28.6 | ECHS1, PCK2, EEF2, KARS, PYCR1, CAT |
| *response to stimulus* | 14.3 | CAT, HSP90AB1, PCK2, |
| *biological regulation* | 9.5 | HSP90AB1, PCK2 |
| *multicellular organismal process* | 4.8 | PCK2 |
| *developmental process* | 4.8 | PCK2 |
| **Molecular function** | | |
| *catalytic activity* | 50.0 | CAT, ECHS1, ALDH2, PCK2, EEF2, KARS, PYCR1 |
| *binding* | 42.9 | CAT, HSPD1, HSP90AB1, PCK2, EEF2, KARS |
| *translation regulator activity* | 7.1 | EEF2 |
| **Cellular component** | | |
| *cell* | 40.0 | CAT, HSP90AB1, PCK2, EEF2, KARS |
| *protein-containing complex* | 18.2 | COL1A2, EIF4H, EEF2, DDX5 |
| *organelle* | 9.1 | PCK2, DDX5 |
| *extracellular region* | 6.7 | COL1A2 |
| *supramolecular complex* | 6.7 | COL1A2 |
| *membrane* | 6.7 | HSP90AB1 |
| **Protein class** | | |
| *metabolite interconversion enzyme* | 54.5 | ALDH2, PCK2, CAT, ECHS1, DLD |
| *translational protein* | 27.3 | EIF4H, EEF2, KARS |
| *chaperone* | 9.1 | HSP90AB1 |
| *extracellular matrix protein* | 9.1 | COL1A2 |

## **Supplementary Table. S5a.**

Presentation of statistical analysis depicted the effect of hypoxia (1% O_2_) on pASCs contractile abilities (statistic refers to Fig.4A in the main body of manuscript).

*Comparisons by group*

| **Day** | **Group** | **lsmean** | **lower.CL** | **upper.CL** | **SE** | **df** | **p.value** |
| --- | --- | --- | --- | --- | --- | --- | --- |
| **0** | **Hypoxia** | 100.0000 | 90.8742 | 109.1258 | 4.2090 | 12.5540 | **1.000** |
| **0** | **Normoxia** | 100.0000 | 91.8376 | 108.1624 | 3.7646 | 12.5540 |  |
| **1** | **Hypoxia** | 58.2690 | 49.1432 | 67.3949 | 4.2090 | 12.5540 | **<0.001** |
| **1** | **Normoxia** | 100.0000 | 91.8376 | 108.1624 | 3.7646 | 12.5540 |  |
| **2** | **Hypoxia** | 42.8587 | 33.7328 | 51.9845 | 4.2090 | 12.5540 | **<0.001** |
| **2** | **Normoxia** | 100.0000 | 91.8376 | 108.1624 | 3.7646 | 12.5540 |  |
| **3** | **Hypoxia** | 37.2995 | 28.1736 | 46.4253 | 4.2090 | 12.5540 | **<0.001** |
| **3** | **Normoxia** | 100.0000 | 91.8376 | 108.1624 | 3.7646 | 12.5540 |  |
| **4** | **Hypoxia** | 30.8720 | 21.7462 | 39.9978 | 4.2090 | 12.5540 | **<0.001** |
| **4** | **Normoxia** | 100.0000 | 91.8376 | 108.1624 | 3.7646 | 12.5540 |  |
| **5** | **Hypoxia** | 28.0120 | 18.8861 | 37.1378 | 4.2090 | 12.5540 | **<0.001** |
| **5** | **Normoxia** | 100.0000 | 91.8376 | 108.1624 | 3.7646 | 12.5540 |  |
| **6** | **Hypoxia** | 25.6654 | 16.5395 | 34.7912 | 4.2090 | 12.5540 | **<0.001** |
| **6** | **Normoxia** | 100.0000 | 91.8376 | 108.1624 | 3.7646 | 12.5540 |  |
| **7** | **Hypoxia** | 24.9167 | 15.7908 | 34.0425 | 4.2090 | 12.5540 | **<0.001** |
| **7** | **Normoxia** | 100.0000 | 91.8376 | 108.1624 | 3.7646 | 12.5540 |  |
| **9** | **Hypoxia** | 26.4375 | 17.3117 | 35.5633 | 4.2090 | 12.5540 | **<0.001** |
| **9** | **Normoxia** | 100.0000 | 91.8376 | 108.1624 | 3.7646 | 12.5540 |  |

| **Day** | **Group** | **lsmean** | **lower.CL** | **upper.CL** | **SE** | **df** | **Day 1** | **Day 2** | **Day 3** | **Day 4** | **Day 5** | **Day 6** | **Day 7** | **Day 9** |
| --- | --- | --- | --- | --- | --- | --- | --- | --- | --- | --- | --- | --- | --- | --- |
| **0** | **Hypoxia** | 100.00 | 90.87 | 109.13 | 4.21 | 12.55 | **<0.001** | **<0.001** | **<0.001** | **<0.001** | **<0.001** | **<0.001** | **<0.001** | **<0.001** |
| **1** | **Hypoxia** | 58.27 | 49.14 | 67.39 | 4.21 | 12.55 |  | **<0.001** | **<0.001** | **<0.001** | **<0.001** | **<0.001** | **<0.001** | **<0.001** |
| **2** | **Hypoxia** | 42.86 | 33.73 | 51.98 | 4.21 | 12.55 |  |  | 0.725 | 0.012 | **<0.001** | **<0.001** | **<0.001** | **<0.001** |
| **3** | **Hypoxia** | 37.30 | 28.17 | 46.43 | 4.21 | 12.55 |  |  |  | 0.549 | 0.113 | 0.017 | 0.009 | 0.033 |
| **4** | **Hypoxia** | 30.87 | 21.75 | 40.00 | 4.21 | 12.55 |  |  |  |  | 0.993 | 0.789 | 0.647 | 0.900 |
| **5** | **Hypoxia** | 28.01 | 18.89 | 37.14 | 4.21 | 12.55 |  |  |  |  |  | 0.998 | 0.988 | 1.000 |
| **6** | **Hypoxia** | 25.67 | 16.54 | 34.79 | 4.21 | 12.55 |  |  |  |  |  |  | 1.000 | 1.000 |
| **7** | **Hypoxia** | 24.92 | 15.79 | 34.04 | 4.21 | 12.55 |  |  |  |  |  |  |  | 1.000 |
| **9** | **Hypoxia** | 26.44 | 17.31 | 35.56 | 4.21 | 12.55 |  |  |  |  |  |  |  |  |
| **0** | **Normoxia** | 100.00 | 91.84 | 108.16 | 3.76 | 12.55 | 1.000 | 1.000 | 1.000 | 1.000 | 1.000 | 1.000 | 1.000 | 1.000 |
| **1** | **Normoxia** | 100.00 | 91.84 | 108.16 | 3.76 | 12.55 |  | 1.000 | 1.000 | 1.000 | 1.000 | 1.000 | 1.000 | 1.000 |
| **2** | **Normoxia** | 100.00 | 91.84 | 108.16 | 3.76 | 12.55 |  |  | 1.000 | 1.000 | 1.000 | 1.000 | 1.000 | 1.000 |
| **3** | **Normoxia** | 100.00 | 91.84 | 108.16 | 3.76 | 12.55 |  |  |  | 1.000 | 1.000 | 1.000 | 1.000 | 1.000 |
| **4** | **Normoxia** | 100.00 | 91.84 | 108.16 | 3.76 | 12.55 |  |  |  |  | 1.000 | 1.000 | 1.000 | 1.000 |
| **5** | **Normoxia** | 100.00 | 91.84 | 108.16 | 3.76 | 12.55 |  |  |  |  |  | 1.000 | 1.000 | 1.000 |
| **6** | **Normoxia** | 100.00 | 91.84 | 108.16 | 3.76 | 12.55 |  |  |  |  |  |  | 1.000 | 1.000 |
| **7** | **Normoxia** | 100.00 | 91.84 | 108.16 | 3.76 | 12.55 |  |  |  |  |  |  |  | 1.000 |
| **9** | **Normoxia** | 100.00 | 91.84 | 108.16 | 3.76 | 12.55 |  |  |  |  |  |  |  |  |

## **Supplementary Table. S5b.**

Presentation of statistical analysis depicted the effect of hypoxia (1% O_2_) on pASCs contractile abilities (statistic refers to Fig.4A in the main body of manuscript).

*Comparisons by days.*

## **Supplementary Table. S6a.**

Presentation of statistical analysis depicted the effect of hypoxia (1% O_2_) on pASCs migration (statistic refers to Fig.4B in the main body of manuscript).

*Comparisons by group*

| **Time [h]** | **Group** | **lsmean** | **lower.CL** | **upper.CL** | **SE** | **df** | **p.value** |
| --- | --- | --- | --- | --- | --- | --- | --- |
| **0** | **Hypoxia** | 100.0000 | 91.1696 | 108.8304 | 4.4310 | 73.2598 | **1.000** |
| **0** | **Normoxia** | 100.0000 | 91.1696 | 108.8304 | 4.4310 | 73.2598 |  |
| **4** | **Hypoxia** | 71.8185 | 62.9881 | 80.6489 | 4.4310 | 73.2598 | **0.193** |
| **4** | **Normoxia** | 80.0495 | 71.2191 | 88.8799 | 4.4310 | 73.2598 |  |
| **8** | **Hypoxia** | 58.1760 | 49.3456 | 67.0064 | 4.4310 | 73.2598 | **0.817** |
| **8** | **Normoxia** | 59.6298 | 50.7994 | 68.4602 | 4.4310 | 73.2598 |  |
| **20** | **Hypoxia** | 27.0331 | 18.2027 | 35.8635 | 4.4310 | 73.2598 | **0.499** |
| **20** | **Normoxia** | 22.7718 | 13.9414 | 31.6022 | 4.4310 | 73.2598 |  |
| **28** | **Hypoxia** | 11.3848 | 2.5544 | 20.2152 | 4.4310 | 73.2598 | **0.624** |
| **28** | **Normoxia** | 8.3042 | -0.5262 | 17.1346 | 4.4310 | 73.2598 |  |
| **48** | **Hypoxia** | -0.0000 | -8.8304 | 8.8304 | 4.4310 | 73.2598 | **1.000** |
| **48** | **Normoxia** | 0.0000 | -8.8304 | 8.8304 | 4.4310 | 73.2598 |  |

## **Supplementary Table. S6b.**

Presentation of statistical analysis depicted the effect of hypoxia (1% O_2_) on pASCs migration (statistic refers to Fig.4B in the main body of manuscript).

*Comparisons by hours.*

| **Time**  **[h]** | **Group** | **lsmean** | **lower.CL** | **upper.CL** | **SE** | **df** | **4 h** | **8 h** | **20 h** | **28 h** | **48 h** |
| --- | --- | --- | --- | --- | --- | --- | --- | --- | --- | --- | --- |
| **0** | **Hypoxia** | 100.00 | 91.17 | 108.83 | 4.43 | 73.26 | **<0.001** | **<0.001** | **<0.001** | **<0.001** | **<0.001** |
| **4** | **Hypoxia** | 71.82 | 62.99 | 80.65 | 4.43 | 73.26 |  | 0.173 | **<0.001** | **<0.001** | **<0.001** |
| **8** | **Hypoxia** | 58.18 | 49.35 | 67.01 | 4.43 | 73.26 |  |  | **<0.001** | **<0.001** | **<0.001** |
| **20** | **Hypoxia** | 27.03 | 18.20 | 35.86 | 4.43 | 73.26 |  |  |  | 0.080 | **<0.001** |
| **28** | **Hypoxia** | 11.38 | 2.55 | 20.22 | 4.43 | 73.26 |  |  |  |  | 0.355 |
| **48** | **Hypoxia** | -0.00 | -8.83 | 8.83 | 4.43 | 73.26 |  |  |  |  |  |
| **0** | **Normoxia** | 100.00 | 91.17 | 108.83 | 4.43 | 73.26 | 0.010 | **<0.001** | **<0.001** | **<0.001** | **<0.001** |
| **4** | **Normoxia** | 80.05 | 71.22 | 88.88 | 4.43 | 73.26 |  | 0.008 | **<0.001** | **<0.001** | **<0.001** |
| **8** | **Normoxia** | 59.63 | 50.80 | 68.46 | 4.43 | 73.26 |  |  | **<0.001** | **<0.001** | **<0.001** |
| **20** | **Normoxia** | 22.77 | 13.94 | 31.60 | 4.43 | 73.26 |  |  |  | 0.128 | 0.002 |
| **28** | **Normoxia** | 8.30 | -0.53 | 17.13 | 4.43 | 73.26 |  |  |  |  | 0.693 |
| **48** | **Normoxia** | 0.00 | -8.83 | 8.83 | 4.43 | 73.26 |  |  |  |  |  |

## **Supplementary Table. S7.**

Presentation of statistical analysis depicted the effect of hypoxia (1% O_2_) on pASCs proliferation assessed by BrdU incorporation (statistic refers to Fig.4C in the main body of manuscript).

|  | **Estimate** | **Std. Error** | **df** | **t value** | **Pr(>\|t\|)** |
| --- | --- | --- | --- | --- | --- |
| **(Intercept)** | 90.88 | 8.80 | 8.6 | 10.329 | **0.000** |
| **Normoxia** | -17.45 | 12.44 | 8.6 | -1.402 | **0.196** |
| **48 h** | -6.38 | 6.36 | 12.0 | -1.003 | **0.336** |
| **72 h** | -4.67 | 6.36 | 12.0 | -0.735 | **0.476** |
| **Normoxia: 48 h** | -6.80 | 8.99 | 12.0 | -0.756 | **0.464** |
| **Normoxia: 72 h** | 2.65 | 8.99 | 12.0 | 0.295 | **0.773** |

## **Supplementary Table. S8.**

Presentation of statistical analysis depicted the effect of hypoxia (1% O_2_) on pASCs proliferation assessed by cells count (statistic refers to Fig.4D in the main body of manuscript).

|  | **Estimate** | **Std. Error** | **t value** | **Pr(>\|t\|)** |
| --- | --- | --- | --- | --- |
| **(Intercept)** | 3.072 | 0.454 | 6.761 | **0.000** |
| **Normoxia** | -0.509 | 0.643 | -0.792 | **0.439** |
| **48 h** | -1.045 | 0.643 | -1.626 | **0.122** |
| **72 h** | -0.526 | 0.694 | -0.758 | **0.459** |
| **Normoxia: 48 h** | 1.094 | 0.909 | 1.204 | **0.245** |
| **Normoxia: 72 h** | 0.166 | 0.946 | 0.175 | **0.863** |

## **Supplementary Table. S9a.**

Presentation of statistic depicted the effect of hypoxia (1% O_2_) on collagen I alpha 2 secretion by pASCs (statistic refers to Fig.4E in the main body of manuscript).

*Summary of mixed-effects model for all data*

|  | **Estimate** | **Std. Error** | **df** | **t value** | **Pr(>\|t\|)** |
| --- | --- | --- | --- | --- | --- |
| **(Intercept)** | 347.75 | 40.0 | 12.9 | 8.702 | **0.000** |
| **Normoxia.(21.%.O2)** | 5.74 | 34.5 | 9.0 | 0.166 | **0.872** |

## **Supplementary Table. S9b.**

Presentation of statistic depicted the effect of hypoxia (1% O_2_) on collagen I alpha 2 secretion by pASCs (statistic refers to Fig.4E in the main body of manuscript).

Summary of mixed-effects model after outliers removal

|  | **Estimate** | **Std. Error** | **df** | **t value** | **Pr(>\|t\|)** |
| --- | --- | --- | --- | --- | --- |
| **(Intercept)** | 331.44 | 26.2 | 10.79 | 12.673 | **0.000** |
| **Normoxia.(21.%.O2)** | -6.53 | 17.0 | 7.12 | -0.385 | **0.712** |

## **Supplementary Figure S2**


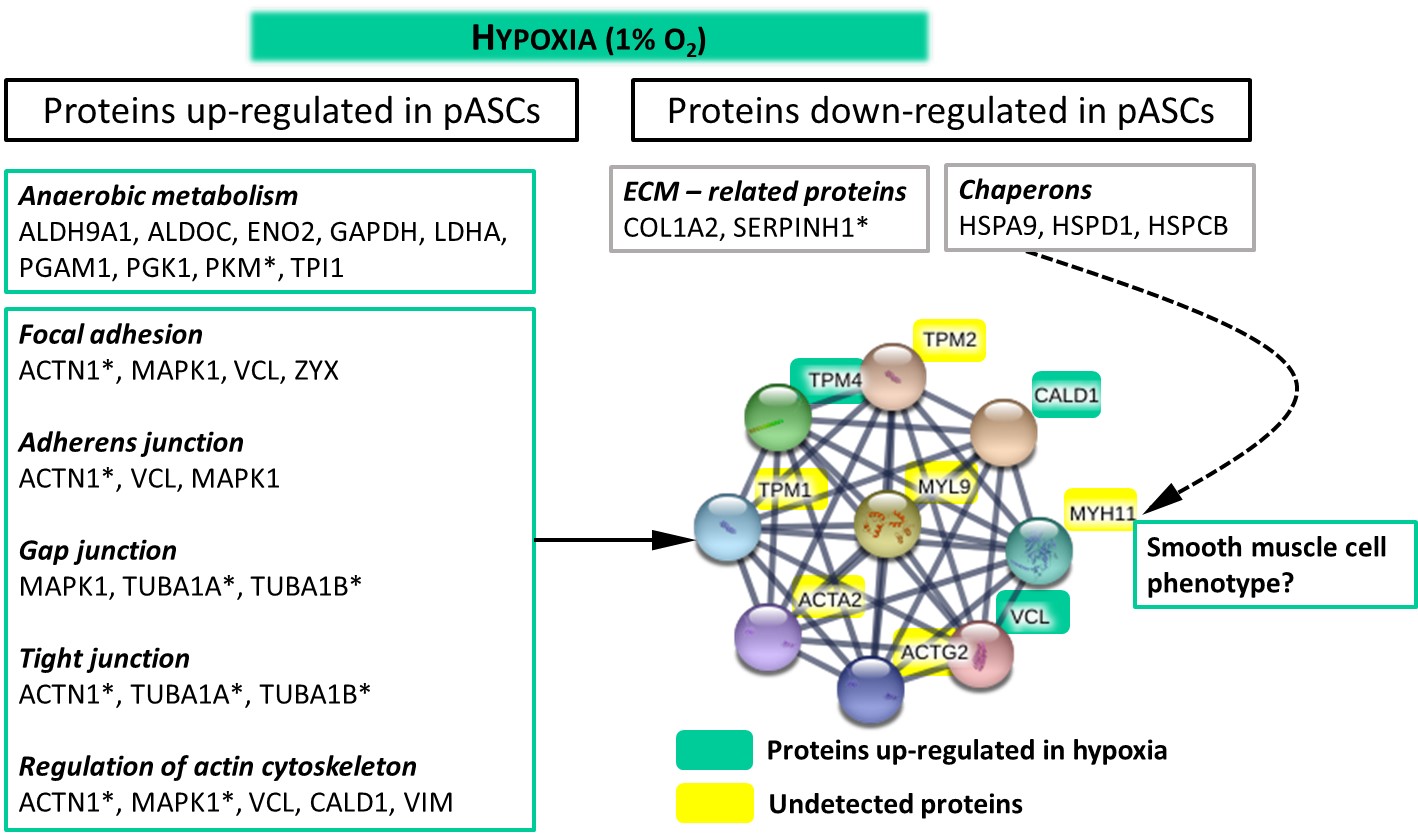


# **Supplementary Figure Legend S2**

Schematic illustration of selected pASCs proteins regulated by hypoxia (1% O_2_) identified in the STRING database (version 11.0). Pathways enriched by proteins up-regulated (left panel) and down-regulated (right panel) following hypoxia were chosen based on their involvement in the cellular responses to hypoxia and wound healing processes, as discussed in the manuscript. Protein-protein interaction networks for pASCs genes with increased expression following hypoxic preconditioning pointed to proteins involved in the smooth muscle cell phenotype and cell contractile abilities (actin, alpha 2, smooth muscle, aorta, ACTA2; myosin regulatory light polypeptide 9, MYL9; myosin-11, MYH11; tropomyosin alpha-1 and -4 chains, TPM1, TPM4). Asterisk (*) indicates proteins that show the expression fold-change ≤ 1.10.

# **Supporting Information Methods**

## **Proteomics**

*Fluorescence labeling of pASCs proteins with CyDyes and 2D-DIGE.* Protein labeling with CyDye DIGE Fluor and 2D electrophoresis were performed as previously described by Kur-Piotrowska et al.[^1^](#_ENREF_1) Briefly, 50 μg of each sample were minimally labeled by incubation with 400 pmol of amine-reactive cyanine dye (Cy3 or Cy5) for 30 min in the dark. The internal standard was generated by combining equal amounts of proteins from each of 8 samples and labeling with Cy2 for a ratio of 50  μg protein to 400  pmol Cy2. The arrangement of the pASC hypoxia and normoxia samples for the 2D-DIGE experiment is shown in the Supplementary Table S10. The samples were loaded onto Immobiline DryStrip gel strips (24 cm, pH 3 to 10 non-linear; GE Healthcare) with passive rehydration (18 h). Isoelectric focusing was performed with an IPGphor isoelectric focusing unit (GE Healthcare), and SDS-PAGE was run using the ETTAN Dalt six electrophoresis unit (GE Healthcare) as described by Słowińska et al.[^2^](#_ENREF_2)

*Image Acquisition and Quantitative Analysis.* The CyDye-labeled gels were analyzed by post-run fluorescence imaging with the use of a Typhoon FLA 9500 instrument (GE Healthcare). Images were analyzed with DeCyder Differential Analysis Software (Version 5.02 software, GE Healthcare). All spots with a *p* value < 0.05 were considered as differentially abundant and used for further analysis. Next, gels were stained with Coomassie Brilliant Blue R-250 and used for manual protein spot picking.

*MALDI TOF/TOF protein identification.* Spots of interests were subjected to reduction, alkylation, and in-gel trypsin digestion as described by Słowińska et al.[^3^](#_ENREF_3) Mass spectra were acquired in the range of 500-3500 m/z, using an MALDI-TOF AutoFlex Speed TOF/TOF mass spectrometer equipped with a Smartbeam II laser (355 nm, Bruker Daltonics) and operating conditions described by Słowińska et al.[^3^](#_ENREF_3) Peptide masses from mass spectrometry analysis and their fragments obtained from MS/MS spectra were combined and submitted to bioanalyses searched using Mascot Server (Matrix Science, London, UK) and the NCBI database. The database search criteria were as follows: enzyme, trypsin; fixed modification, carbamidomethylation (C); and variable modifications, oxidation (M) peptide mass tolerance of 200 ppm, fragment mass tolerance of 0.7 Da, and one missed cleavage allowed. The search results were filtered with a significance threshold of *p* < 0.05 and a MASCOT ion score cut-off ≥30.

**Supplementary Table S10.**

The pASC arrangement for a 2D-DIGE experiment in four biological replicates. The internal standard was generated when equal amounts of protein from each of the 8 samples were combined.

| Gel number | Cy2 (50µg) | Cy3 (50µg) | Cy5 (50µg) |
| --- | --- | --- | --- |
| Gel 1 | Internal Std | Hypoxia1 | Normoxia 3 |
| Gel 2 | Internal Std | Normoxia 1 | Hypoxia3 |
| Gel 3 | Internal Std | Hypoxia 2 | Normoxia 4 |
| Gel 4 | Internal Std | Normoxia 2 | Hypoxia 4 |

## **RNA isolation and real-time PCR**

Total RNA was purified from pASCs cultured under hypoxia (n=8) or normoxia (n=8) using TRIzol**^®^** Reagent (Thermo Fisher Scientific). Genomic DNA was removed from RNA samples using DNase I Amplification Grade kits (Sigma-Aldrich Co.). Reverse transcription was performed using High-Capacity cDNA Reverse Transcription Kits with RNase Inhibitor (Applied Biosystems by Thermo Fisher Scientific) according to the manufacturer's specifications. Endogenous mRNA expression levels were measured with Custom TaqMan® Gene Expression Assays (Applied Biosystems by Thermo Fisher Scientific) using an ABI ViiA™ 7 sequence detection system (Applied Biosystems by Life Technologies). Gene names and primer-probe sets information are presented in Supplementary Tab. S2. PCR cycling and detection were performed using 10 min at 95°C, 45 cycles of 15 s at 95°C, and 1 min at 60°C. All results were normalized to *HPRT11* content and analyzed using the PCR Miner algorithm[^4^](#_ENREF_4).

**Supplementary Table S11.**

Gene names and primer-probe sets used in real time PCR.

| **Gene Symbol** | **TaqMan® primer and probe sets ID** |
| --- | --- |
| *P4HA1* | Ss03388847_m1 |
| *LDHA* | Ss04246521_m1 |
| *ENO1* | Ss06866694_g1 |
| *PGK1* | Ss03389144_m1 |
| *ALDOC* | Ss04325913_g1 |
| *HSP70.2* | Ss03392270_g1 |
| *CAT* | Ss04323025_m1 |
| *HSPD1* | Ss01036749_m1 |
| *EPRS* | Ss03373859_m1 |
| *GLUD1* | Ss04327630_m1 |
| *ALDH2* | Ss03388488_m1 |
| *PCK2* | Ss03373733_m1 |
| *COL1A2* | ss03375009_u1 |
| *HPRT1* | Ss03388274_m1 |

## **Collagen gel contraction assay**

pASCs suspensions (1.0×10^5^ cells/400 μL; n = 5) at passage 1 were mixed in 200 μL of a solution of rat tail tendon collagen type I (5 mg/mL, Cultrex). Next, 500 μL of the mixture was added to each well of a 24-well plate, neutralized with 5 μL of 1 M NaOH, and allowed to polymerize. After solidification, gel matrices were overlaid with 500 μL of ADSC-BM with 10% FBS, L-glutamine, and antibiotics. The floating gels were incubated for 24 h at 37°C under normoxic (21% O_2_, 5% humidified CO_2_) or hypoxic (1%, O_2_, 5% humidified CO_2_) conditions for 9 days. Collagen lattices were photographed with an Olympus Digital Camera E-M10MarkII, and the areas were measured with ImageJ software (National Institutes of Health). Gel contraction was calculated as the percentage of the initial gel area at time 0, which was considered to be 100%.

## ***In vitro* wound migration assay**

pASCs (p = 1; n = 8) were plated in duplicate in 12-well plates at a density of 5.0×10^5^ cells per well and allow to grow until they reached confluence. To prevent proliferation, cells were incubated for 3 h with mitomycin C (10 μg/mL). Next, cell monolayers were scraped in a straight line through the center of the well with a 200-μL pipet tip. The cultures were incubated for 24 h at 37°C under normoxic (21% O_2_, 5% humidified CO_2_) or hypoxic (1%, O_2_, 5% humidified CO_2_) conditions in ADSC-BM with 10% FBS, L-glutamine, and antibiotics. Images were recorded with an Olympus microscope (IX51) equipped with an Olympus digital camera (XC50) and analyzed with ImageJ. Representative images of scratched areas were photographed, and the distance between the reduction of edge distance was measured. The distance of scratch closure at 0 h was considered to be 100%.The scratched areas were monitored until closure (at 0, 4, 8, 20, 28 and 48 h time points)

# **Supplementary References**

1 Kur-Piotrowska, A. *et al.* Foxn1 expression in keratinocytes is stimulated by hypoxia: further evidence of its role in skin wound healing. *Scientific reports* **8**, 5425, doi:10.1038/s41598-018-23794-5 (2018).

2 Slowinska, M., Kozlowski, K., Jankowski, J. & Ciereszko, A. Proteomic analysis of white and yellow seminal plasma in turkeys (Meleagris gallopavo). *Journal of animal science* **93**, 2785-2795, doi:10.2527/jas.2015-8912 (2015).

3 Slowinska, M. *et al.* 2D-DIGE proteomic analysis reveals changes in haemolymph proteome of 1-day-old honey bee (Apis mellifera) workers in response to infection with Varroa destructor mites. *Apidologie* **50**, 632-656, doi:10.1007/s13592-019-00674-z (2019).

4 Zhao, S. & Fernald, R. D. Comprehensive algorithm for quantitative real-time polymerase chain reaction. *Journal of computational biology : a journal of computational molecular cell biology* **12**, 1047-1064, doi:10.1089/cmb.2005.12.1047 (2005).
